# Mitochondrial Haplogroup Influences Motor Function in Long-Term HIV-1-Infected Individuals

**DOI:** 10.1371/journal.pone.0163772

**Published:** 2016-10-06

**Authors:** Ashley Azar, Kathryn Devlin, Joshua Chang Mell, Tania Giovannetti, Vanessa Pirrone, Michael R. Nonnemacher, Shendra Passic, Katherine Kercher, Jean W. Williams, Jeffery M. Jacobson, Brian Wigdahl, William Dampier, David J. Libon, Christian Sell

**Affiliations:** 1 Department of Pathology, Drexel University College of Medicine, Philadelphia, Pennsylvania, United States of America; 2 Department of Psychology, Temple University, Philadelphia, Pennsylvania, United States of America; 3 Department of Microbiology and Immunology, Drexel University College of Medicine, Philadelphia, Pennsylvania, United States of America; 4 Center for Genomic Sciences, Institute for Molecular Medicine and Infectious Disease, Drexel University College of Medicine, Philadelphia, Pennsylvania, United States of America; 5 Center for Molecular Virology and Translational Neuroscience, Institute for Molecular Medicine and Infectious Disease, Drexel University College of Medicine, Philadelphia, Pennsylvania, United States of America; 6 Department of Medicine, Lewis Katz School of Medicine, Temple University, Philadelphia, Pennsylvania, United States of America; 7 Department of Neuroscience, Lewis Katz School of Medicine, Temple University, Philadelphia, Pennsylvania, United States of America; 8 Sidney Kimmel Cancer Center, Thomas Jefferson University, Philadelphia, Pennsylvania, United States of America; 9 Department of Geriatrics and Gerontology, New Jersey Institute for Successful Aging, School of Osteopathic Medicine, Rowan University, Stratford, New Jersey, United States of America; Universidade do Porto Instituto de Patologia e Imunologia Molecular, PORTUGAL

## Abstract

Evolutionary divergence of the mitochondrial genome has given rise to distinct haplogroups. These haplogroups have arisen in specific geographical locations and are responsible for subtle functional changes in the mitochondria that may provide an evolutionary advantage in a given environment. Based on these functional differences, haplogroups could define disease susceptibility in chronic settings. In this study, we undertook a detailed neuropsychological analysis of a cohort of long-term HIV-1-infected individuals in conjunction with sequencing of their mitochondrial genomes. Stepwise regression analysis showed that the best model for predicting both working memory and declarative memory were age and years since diagnosis. In contrast, years since diagnosis and sub-haplogroup were significantly predictive of psychomotor speed. Consistent with this, patients with haplogroup L3e obtained better scores on psychomotor speed and dexterity tasks when compared to the remainder of the cohort, suggesting that this haplogroup provides a protective advantage when faced with the combined stress of HIV-1 infection and long-term antiretroviral therapies. Differential performance on declarative memory tasks was noted for individuals with other sub-L haplogroups, but these differences were not as robust as the association between L3e and psychomotor speed and dexterity tasks. This work provides evidence that mitochondrial haplogroup is related to neuropsychological test performance among patients in chronic disease settings such as HIV-1 infection.

## Introduction

Therapy for human immunodeficiency virus type 1 (HIV-1) infection has altered the clinical course of disease such that infected individuals are now afflicted with a long-term chronic illness rather than an acute terminal disease. Concomitant with this shift in the disease’s natural history are changes in the demographics of the HIV-1-infected population. These changes manifest as an extended survival linked with the advent of combination antiretroviral therapies (cART) [1, 2]. One result of the success of cART is that the HIV-1-infected population has shifted from a population below age 45 years in 1996 to an HIV-1-infected population, with ~50% over 50 years of age in 2015 [3]. While the lifespan of HIV-1-infected patients has increased, it has also become apparent that these individuals have an increased incidence of early-onset age-related neurodegenerative diseases such as Alzheimer’s disease, Parkinson’s disease, and other dementia disorders [4]. The increased risk of neurocognitive decline may be due to the effect of HIV, the long-term side effects of cART therapies, or an interaction between both of these factors.

Thus, despite the benefits of cART therapy, HIV-1-infected individuals often present with serious neuropsychological deficits [5, 6]. For example, a significant co-morbidity associated with HIV-1 infection is HIV-1-associated neurocognitive disorder (HAND), a collective term used to denote the neurocognitive complications exhibited by HIV-1-infected individuals. Patients with HAND may present early signs of central nervous system (CNS) involvement such as psychomotor slowing, impaired encoding/ retrieval on declarative memory tests, and difficulties on executive tests including problems with prospective planning, mental control, and mental manipulation. Gait disturbances, falls, decreased manual dexterity, and neuropsychiatric problems including apathy or depression have also been described [7]. Past research suggests significant heterogeneity in the neuropsychological/neuropsychiatric impairment of people with HAND [8–10]. Although the biological basis for this heterogeneity is not clear, it is likely that an individual’s genetic makeup contributes to these differences.

Although the majority of genetic variation occurs within the nuclear genome, a significant amount of genetic variation also occurs in the mitochondrial genome. Evolutionary pressures have shaped the mitochondrial genome to include regional variations, known as haplogroups [11]. Among other metabolic changes, these mitochondrial haplogroups can shift mitochondrial energy output between ATP production and heat production by altering the coupling efficiency of the electron transport chain in response to environmental pressures, potentially providing distinct evolutionary advantages in different environmental niches [12]. Differences in coupling efficiency create a unique background in each mitochondrial haplogroup causing a distinct interaction with environmental toxins and nuclear influences. For example, a specific interaction between mutations causing Leber's Hereditary Optic Neuropathy and mitochondrial haplogroups indicates that haplogroup J exacerbates this disease phenotype [13]. Importantly for the present study, neural complications of HIV/cART such as peripheral neuropathy have been shown to be more prevalent in patients with the mitochondrial haplogroup L0a2 while patients with haplogroup L2a have a reduced risk for peripheral neuropathy [14]. Other examples include the findings that haplogroups J and UK are underrepresented in Parkinson’s disease populations, suggesting some protection from the disorder, and that haplogroup T is underrepresented in Alzheimer’s patients [15, 16]. Cybrid analysis (transfer of mitochondrial variants onto a common nuclear genetic background) revealed that cells harboring mitochondria of the L haplogroup produced lower levels of ROS (reactive oxygen species) and decreased ATP turnover rates than those harboring the H haplogroup [17]. Thus, accumulating evidence suggests that mitochondrial DNA variation alters an individual’s susceptibility to disease and supports the possibility that mitochondrial variation could play a role in the differential susceptibility of HIV-1 patients to HIV-associated co-morbidities.

Recent research has begun to analyze the impact of mitochondrial genetics on HIV-associated neurologic co-morbidities. For example, the relationship between mitochondrial haplogroup and sensory-neuropathy has been examined in patients from the CNS HIV Anti-Retroviral Therapy Effects Research (CHARTER) study. These data suggests that single nucleotide polymorphisms (SNPs) associated with haplogroup L1c and J may be associated with a decreased prevalence of this co-morbidity [14]. More recently the same research group released results showing that in a cohort of Hispanic individuals, the typical Native-American haplogroup B exhibited lower global deficit scores (indicating better neurocognitive performance), suggesting an influence of mitochondria on neuropsychological functioning [18].

In order to further examine the potential relationship(s) between mitochondrial variation and HAND, we performed a detailed neuropsychological evaluation assessing psychomotor speed and dexterity functioning, declarative memory, and executive control/working memory in a subgroup of the Drexel Medicine CNS AIDS Research and Eradication Study (CARES) cohort, as well as a complete sequence analysis of their mitochondrial genomes. The Drexel Medicine CARES cohort is a continually expanding cohort of 557 patients, which spans approximately 2179 longitudinal visits. This analysis allowed us to identify genetic variation in the mtDNA that may be associated with these neurocognitive functions. These results will further evaluate the hypothesis that for patients with chronic HIV-1 infection, specific mitochondrial haplogroups may be associated with specific patterns of neuropsychological strengths and weaknesses.

## Methods

### Patient DNA preparations

DNA was isolated from PBMC (peripheral blood mononuclear cells) using Qiagen extraction methods, as described by the manufacturer. Mitochondrial DNA was then amplified from total DNA using mitochondrial genome specific primers and utilizing long-range PCR procedures as described by the manufacturer (Qiagen) [19]. mtDNA was amplified in three overlapping fragments. Fragment loci: 6511–10622, 6739–16488, 151–10360. After amplification small amounts of PCR product were run on an agarose gel to confirm proper single band amplification, and PCR products were then purified using a Qiagen PCR clean up kit. Purified mtDNA fragments were quantified using Nanodrop 1000. For each patient the three amplified mtDNA fragments were pooled at equi-molar concentrations prior to sequencing library construction.

### Sequencing

Pooled mtDNA amplicons for each patient were converted to sequencing libraries using Nextera XT prep procedures for tagmentation, amplification and purification as specified in Illumina protocols. Sequencing preps were validated using a bioanalyzer (Agilent 2100 Bioanalyzer) and quantified using a fluorometer (BioTek instruments INC. FLx800) with the Quant-iT dsDNA Assay procedures as described by the manufacturer (Invitrogen). Processed patient mtDNA libraries were then pooled and sequenced using either MiSeq (2x150 nt paired ends) or NextSeq (2x150 nt paired ends) specifications (Illumina).

### Data Analysis

Raw data processing and demultiplexing of NextSeq data used bcl2fastq (MiSeq provided FastQ automatically). BWA-MEM (v0.7.10) was used to align sequencing reads to the revised Cambridge reference sequence (rCRS) [20]. Samtools mpileup (v0.1.19) and bcftools (v2.22.0) were used for variant calling [21]. GATK (v3.3–0) was used to compile consensus FASTA sequences for each patient [22]. Haplogroups were assigned using FASTA files uploaded to the Haplofind.unibo.it website, Haplogroup assignments for each patient are listed in S3 Table. R-Studio (v0.98.1091) and SPSS (v22) were used for statistical analysis and further data manipulation. Specific R packages used include SNPRelate for PCA analysis of genomic data [23–25].

### Data deposition

Sequence data were deposited at the NCBI SRA under BioProject SRA accession: SRP074574 as BAM files. BioSample accessions are in S4 Table.

### Drexel Medicine CNS AIDS Research and Eradication Study (CARES) Cohort

To date, 557 HIV-1-infected patients were recruited from the Philadelphia area to the Drexel Medicine CNS AIDs Research and Eradication Study (CARES) cohort (Drexel University College of Medicine IRB protocol number 1201000748, Principal Investigator, Brian Wigdahl). All patient samples were collected under the auspices of protocol 1201000748 through written consent. The work described in this manuscript was specifically reviewed and approved by the Drexel University Institutional Review Board. This cohort follows patients longitudinally, and to date there are 2179 total visits. 194 patients were administered a comprehensive neuropsychological evaluations, and of those patients a sub-cohort of 157 patients were subjected to full mtDNA sequencing [aged 26–73 (M = 51.52, SD = 8.06), including 105 men (68.1%)]. Participants were predominantly self identified African-American (N = 145, 92.4%), with an average duration of infection of 18.79 years (SD = 7.72). Nearly all participants were being treated with cART at the time of visit (~95%), were clinically stable, had undetectable viral load (<20 copies/ml; 64%) and had a mean current CD4 of 673.48 cells/ul (SD = 364.34). Demographic information for this sub-cohort is displayed in Table 1.

**Table 1:** Drexel Medicine CARES cohort descriptive data for overall cohort, L haplogroups, non-L haplogroups, L3e haplogroups and non-L3e haplogroups.

	Overall (N = 157)	L Haplogroup (N = 128)	Non-L Haplogroups (N = 29)	L3e Sub-Haplogroup (N = 29)	Non-L3e Sub-Haplogroups (N = 128)
**Mean Age (range)**	51.85 (27–73)	51.79 (27–73)	52.14 (27–70)	51.52 (33–73)	51.92 (27–71)
**Female**	50 (33.1%)	44 (34.4%)	9 (31.0%)	14 (48.3%)	39 (29.8%)
**African Ancestry**	145 (92.4%)	123 (96.1%)	22 (75.9%)	27 (93.1%)	118 (92.2%)
**Initial Mean CD4 (cells/uL) (SD)**	379.26 (298.66)	371.22 (294.02)	415.03 (321.45)	332.29 (276.05)	389.38 (303.36)
**Nadir Mean CD4 (cells/uL) (SD)**	230.78 (206.25)	224.97 (209.58)	256.62 (193.22)	186.71 (219.08)	240.27 (203.26)
**Latest Mean CD4 (cells/uL) (SD)**	673.48 (364.34)	665.34 (384.24)	709.69 (260.22)	618.68 (338.02)	685.28 (369.93)
**Initial Viral Load (copies/mL) (SD)**	104731.61 (420372)	118147.58 (464637)	42922.29 (76765)	260597.68 (887576)	70900.21 (206704)
**Peak Viral Load (copies/mL) (SD)**	256900.99 (744920)	240389.98 (548195)	332969.54 (1331970)	579099.79 (1014128)	186966.36 (657050)
**Latest Viral Load (copies/mL) (SD)**	5010.03 (45887.3)	6063.33 (50595)	157.32 (510.76)	22981.64 (107491)	1109.21 (6557)
**Mean Years on HAART (SD)**	12.12 (7.66)	11.70 (7.57)	14.00 (7.96)	10.10 (7.63)	12.56 (7.64)
**Mean Years Since Diagnosis (SD)**	18.79 (7.72)	18.49 (7.75)	20.17 (7.53)	16.45 (7.77)	19.31 (7.64)
**Mean motor score (SD)**	43.30 (7.59)	43.18 (7.56)	43.88 (7.84)	47.41 (7.37)	42.46 (7.38)
**Mean working memory score (SD)**	47.13 (8.31)	47.03 (8.37)	47.55 (8.19)	46.52 (6.74)	47.27 (8.65)
**Mean declarative memory score (SD)**	40.00 (10.93)	39.38 (10.74)	42.81 (11.5)	40.90 (9.40)	39.81 (11.26)

**Fig 1:**
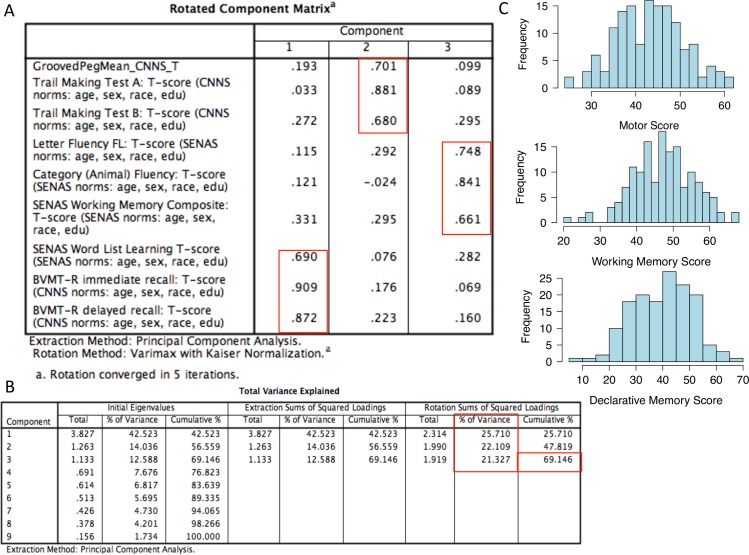
Principle component analysis identifies functional groups among neurocognitive tests. A) Rotated component matrix of norm-derived neuropsychological test scores used to determine neuropsychological composite scores. Component 1 = declarative memory; component 2 = motor; component 3 = working memory. Red boxes show groupings for each component determined by the PCA. B) The nine neurocognitive evaluation scores were compiled in SPSS. Total variance explained by each component is contained under the 3 columns listed under Initial Eigenvalues. Components identified as explaining the maximum amount of variance in the data are listed under Extraction Sums of Squared Loadings. Total amount of variance in the model explained by each of these 3 components after varimax rotation is listed under Rotation Sum of Squared Loadings. Total variance explained by the PCA and variance explained by each component of the PCA is outlined in red boxes. C) Histograms showing that the three composite scores were normally distributed.

Table of demographic, HIV and neurocognitive data with means and standard deviations (SD). Neurocognitive data derived from Fig 1 are listed as: motor score, working memory and declarative memory and are based on published normative data [26, 27]. African ancestry was determined via personal identification.

### Neurocognitive Assessment/ Principal Component Analysis (PCA)

Neuropsychological performance was assessed with nine variables from seven tests selected for their strong psychometric properties, their validity in detecting HIV-associated neurocognitive disorders (HAND), and the availability of demographically appropriate norms. All raw test scores were converted to demographically corrected T-scores (mean = 50; standard deviation = 10) using published normative data stratified by age, education, gender, and population group /ethnicity with higher scores indicating better performance [26, 27]. In each figure, the T-score mean of 50, which reflects average-level performance, is represented by a red dotted line.

### Psychomotor speed and dexterity

#### Grooved Pegboard Test [26, 28]

On this test participants were asked to pick up odd-shaped pegs and fit them into corresponding holes. The dependent variable was time to completion for all test items averaged for both the dominant and non-dominant hands.

#### Trail Making Tests- Parts A and B [26, 29]

For Trail Making Test- Part A participants were asked to draw a line connecting numbers from 1–22. For The Trail Making Test- Part B participants were asked to draw a line alternating between numbers and letters (e.g., 1-A-2-B). Two dependent variables were obtained—time to completion for Part A and time to completion for Part B.

### Declarative memory

#### Brief Visuospatial Memory Test–Revised [26, 30]

Participants were shown a page consisting of six geometric figures for 10 seconds and are asked to draw the figures from memory immediately or after a 20 minute filled delay. Three immediate free recall test trials were administered followed by a long free recall test trial and drawings were assigned points for accuracy. Two dependent variables were obtained, including the total number of points obtained on the three immediate free recall trials and the total number of points obtained on the delay free recall trial.

#### Spanish and English Neuropsychological Assessment Scales (SENAS) Verbal Serial List Learning Test [27]

This test consisted of a 12-word list. Five immediate free recall test trials were administered followed by a delay free recall test trial. A single dependent variable was obtained for the total words recalled across the five immediate free recall test trials and the delay free recall trial.

### Executive control/ working memory

#### SENAS Letter Fluency Test [27]

On this test participants were given 60s to generate words that begin with the letters ‘F’ and ‘L’ excluding proper nouns. The dependent variable was the total words recalled.

#### SENAS Semantic Fluency Test [27]

On this test participants were given 60s to generate the names of animals. The dependent variable was the total number of correct responses.

#### SENAS Working Memory Index [27]

The SENAS Working Memory Index includes four tasks: 1) Digit Span Backwards- participants are read a series of numbers to repeat in reverse order; 2) Visual Span Backwards–participants point to dots on a page in reverse order from that presented by the examiner; 3) List Sorting I–participants are read a list of animals and fruits that they must repeat back in order of size, from smallest to largest; 4) List Sorting II- participants are read a list of animals and fruits that they must repeat back first by category and then in order of size (i.e., fruits first, sorted from smallest to largest, and then animals in order from smallest to largest). The dependent variable is the total number of correct trials summed across all four tasks.

#### Global Deficit Score

The Global Deficit Score (GDS) was calculated to reflect overall level of cognitive impairment. Each of the nine test scores was transformed to a score ranging from zero to five as follows: T scores >40 = 0 (no impairment); T score 35–39 = 1; T score 30–34 = 2; T score 25–29 = 3; T score 20–24 = 4; T score < 19 = 5 (severe impairment). Transformed scores were averaged into a total GDS, with higher scores reflecting greater overall impairment [31].

### Statistical Analyses

In addition to the calculation of the GDS, the nine neuropsychological variables were reduced to three neuropsychological composite scores (psychomotor speed/dexterity; declarative memory; working memory/executive control), l debased on the results of a PCA (see results section). The three neuropsychological composites were analyzed in conjunction with mitochondrial haplogroup to assess relationship to neuropsychological functioning. Initial haplogroup analysis used the R package, SNPRelate to perform a PCA on the mitochondrial genomic data. Haplogroup information from Haplofind was then used as an input to color code individual patient samples. After assigning haplogroups and breaking the L haplogroups further into subgroups seven sub-haplogroups reached a minimum 5% frequency threshold within our study population and therefore were included in subsequent analyses (44). Kruskal-Wallis tests were used to examine differences between haplogroups on the three neuropsychological composite scores due to unequal sample sizes. Mann-Whitney U tests were used to compare each sub-haplogroup to the remainder of the cohort. Finally, stepwise linear regression was performed using SPSS and included the following independent variables: all sub-haplogroups that represent at least 5% of the cohort input as binary variables (L1b, L1c, L2a, L3b, L3d, L3e and Non-Ls), years on cART, years since diagnosis, age, and gender. Three separate stepwise linear regressions were performed for each neuropsychological composite score/dependent variable: psychomotor speed and dexterity, declarative memory, executive/ working memory.

## Results

### Demographic and Clinical Characterization of the Haplogroups

In order to evaluate our main hypothesis that mitochondrial variation can affect HAND phenotypes, we first compiled and analyzed our neurocognitive data with respect to haplogroup for the set of 157 patients. Demographics of the patient population are presented in Table 1. There were no significant differences in HIV-related parameters by haplogroup (CD4 counts, years on therapy, viral load), and the majority of patients in all haplogroups reported African American ancestry (96.1% of L haplogroup and 75.9% of non-L).

### Reduction of Neuropsychological Data and Neuropsychological Characterization of the Groups

The Spanish and English Neuropsychological Assessment Scales (SENAS) neuropsychological protocol described below in the methods was selected, in part, because of the availability of normative data stratified by age, education, population group, and sex [26, 27]. Demographically adjusted T-scores for the nine neuropsychological parameters were reduced to three neurocognitive composites based on the results of a principal component analysis (PCA) with varimax rotation (Fig 1A). These 3 composites accounted for 69.15 percent of variance (Fig 1B). As displayed in Fig 1B Psychomotor Speed and Dexterity (Pegboard, Trails A, Trails B) accounted for 22.11% of the variance. Executive Function/Working Memory measures (letter fluency, semantic fluency, SENAS Working Memory Index) accounted for 21.33% of the variance. Finally, Declarative Memory measures (BVMT and SENAS Verbal Word List Learning) accounted for 25.71% of the variance (Fig 1A and 1B). Based on the results of this PCA, the nine SENAS neuropsychological variables were reduced to three composite indices by averaging the T-scores tests within each factor (psychomotor speed and dexterity composite = 43.30 ± 7.59; declarative memory composite = 40.00 ± 10.93; executive function/working memory composite = 47.13 ± 8.31) (Table 1). Histograms showing the normal distribution of each composite score are shown in Fig 1C.

Mean neuropsychological composite scores shown in Table 1 demonstrate that overall the groups performed within the average (i.e., T-scores between 57 and 43) and the low average (i.e., T-scores between 42 and 36) range on the three neuropsychological composite scores. Also, there was no significant difference between the groups on the three neuropsychological composites.

### Haplogroup Principal Component Analysis

**Fig 2:**
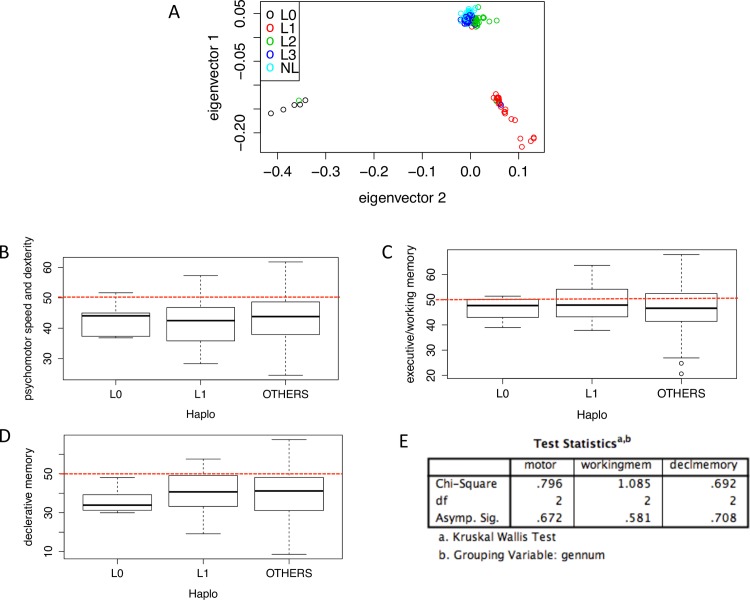
Principle component analysis on patient genotype information. A) Principle component analysis revealed 3 genotype groupings. Color-coding showed patients clustering in 3 major groups L0, L1 and all other haplogroups. Performed using SNPRelate R package B-D) Box whisker plot grouping haplogroups as follows L0 vs L1 vs L2+L3+NL vs. the 3 complied neuropsychological composite scores. E) Statistical analysis between the 3 groups was performed using SPSS and Kruskal-Wallis testing (p<0.05). Red dotted lines indicate mean T-scores.

**Fig 3:**
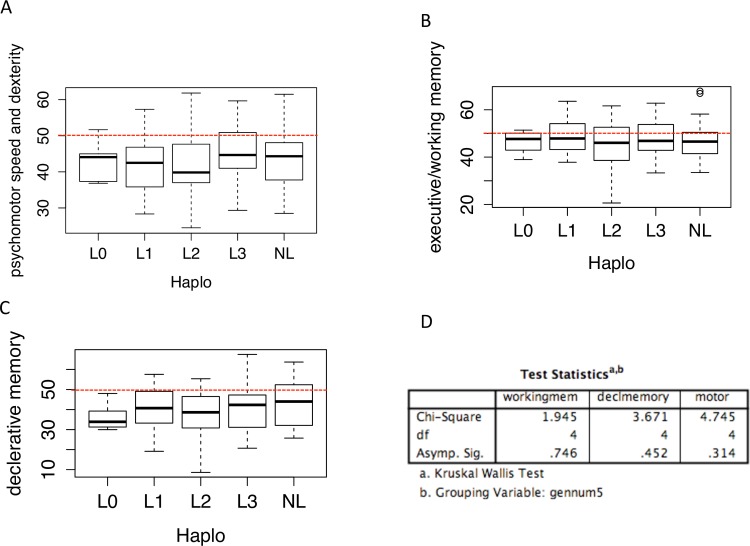
Major haplogroups show no association with neurocognitive performance. A-C) Box whisker plots of the haplogroups against the three compiled neuropsychological composite scores: A- Psychomotor speed B- Working memory C-Declarative memory. Red dotted lines indicate average T-scores. D) SPSS table reporting p-values for Kruskal-Wallis testing.

In order to evaluate the influence of haplogroup on neurocognition, the three PCA- derived neuropsychological composite scores were analyzed in conjunction with mitochondrial haplogroup. Using the R package SNPRelate, we performed a PCA on the mitochondrial genomic data. Haplogroup information assigned using Haplofind [32] was then used as an input to color code individual patient samples (Fig 2A). This mitochondrial genomic PCA identified 3 distinct genetic groups in our cohort; L0, L1 and a combined group of L2+L3+Non-Ls (Fig 2B–2D). These groups are consistent with the ancient origin of the L0 and L1 haplogroups relative to the L2 and L3 split at ~80–60 ka [33–35]. Subsequent analyses of the differences in neurocognitive scores between the three major haplogroups revealed no significant differences (Kruskal-Wallis testing: psychomotor speed and dexterity p-value = 0.67, declarative memory p-value = 0.71, executive function/working memory p-value = 0.58) (Fig 2E). Further analysis, testing the difference in neurocognitive scores between the major L groupings, L0, L1, L2, L3 and non-L haplogroups showed no statistically significant difference between groups (Kruskal-Wallis testing: psychomotor speed and dexterity p-value = 0.31, declarative memory p-value = 0.45, executive function/working memory p-value = 0.75) (Fig 3A–3D).

### Sub-Haplogroup Analysis

**Fig 4:**
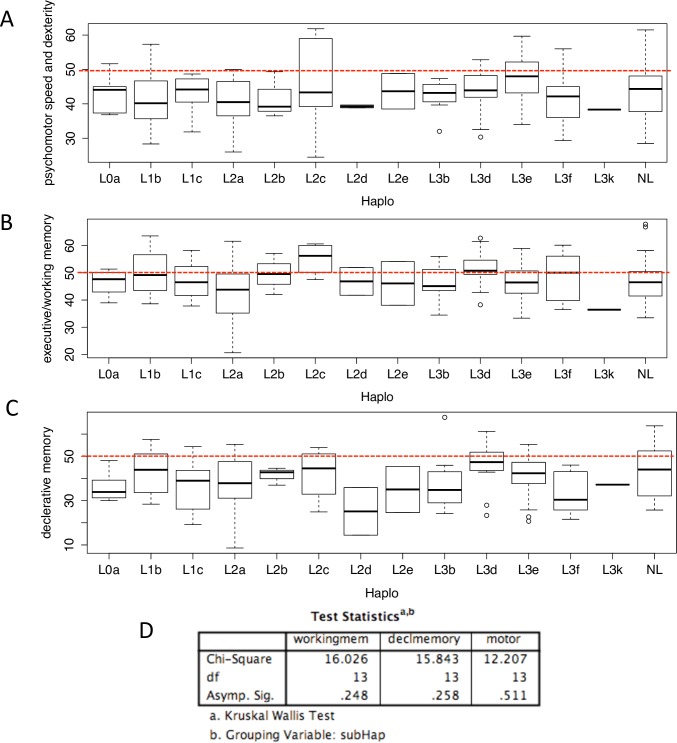
Sub-haplogroups show no association with neurocognitive performance when compared to one another. A-C) Box whisker plots of the sub-haplogroups against the three compiled neuroscores: A- Psychomotor speed B- Working memory C-Declarative memory. Red dotted lines indicate the mean T-scores. D) SPSS table reporting p-values for Kruskal-Wallis testing.

Because we observed no difference in the major L vs non-L haplogroups, we further defined sub-L haplogroups that dominated the patient cohort to examine potential patterns associations with neuropsychological performance on a finer level. The initial analysis examined differences between the major sub-L haplogroups present in the cohort (L0a, L1b, L1c, L2a, L2b, L2c, L2d, L2e, L3b, L3d, L3e, L3f and all non-L haplogroups) (Fig 4A–4D). This analysis revealed no statistical differences in neurocognitive function overall (Kruskal-Wallis testing: psychomotor speed and dexterity p-value = 0.51, declarative memory p-value = 0.26, executive function/working memory p-value = 0.25) However, an analysis separating the sub-L haplogroups as comparators to the remainder of the cohort revealed two sub-L haplogroups that did significantly differ from the rest of the cohort. Table 2 shows p-values for all comparisons and the number of patients in each sub-group; no values are shown for sub-haplogroups that represent less than 5% of the cohort.

**Table 2:** Sub-haplogroup associations with neurocognitive performance when segregated from the cohort as a whole.

Sub-L-Haplogroup	Number of people	Motor Function p-value	Working Memory p-value	Declarative Memory p-value	GDS
**L0a**	6	—	—	—	—
**L1b**	15	0.306	0.177	0.205	0.842
**L1c**	11	0.909	0.980	0.240	0.735
**L2a**	26	0.100	0.025	0.259	0.022
**L2b**	3	—	—	—	—
**L2c**	5	—	—	—	—
**L2d**	2	—	—	—	—
**L2e**	2	—	—	—	—
**L3b**	8	0.704	0.713	0.354	0.899
**L3d**	12	0.811	0.063	0.060	0.203
**L3e**	24	0.004	0.650	0.629	0.218
**L3f**	7	—	—	—	—
**NL**	28	0.774	0.847	0.168	0.343

Specific sub-haplogroups are listed with a breakdown including number of patients of each sub-haplogroup and the p-values for all Mann-Whitney U segregation comparisons for the three neuropsychological composite scores derived from the PCA analysis as well as p-values for comparisons with GDS (Global Deficit Score), shown in blue text. Total number of patients listed is less than total number of patients in Table 1 due to missing neurocognitive values. Sub-haplogroups, which contain fewer than 5% of the total cohort, were excluded from the analysis.

**Fig 5:**
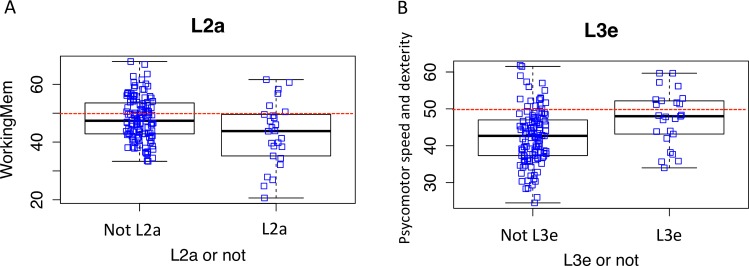
Sub-haplogroups L2a and L3e show statistically significant association with neurocognitive performance when segregated from the cohort as a whole. A-B) Box whisker plots of 2 sub-haplogroups (L2a and L3e) significantly associated with performance on at least one neuropsychological composite score with respect to the rest of the cohort. Working memory (A) and psychomotor speed and dexterity (B.) Mann-Whitney U statistical testing (p-value <0.05). Red dotted lines indicate mean values expressed as T-scores.

Individuals of L2a had a lower executive/working memory composite score than the rest of the cohort (p = 0.025) (Fig 5A). Additionally, individuals of L3e haplogroups had a higher psychomotor speed and dexterity composite score than the rest of the cohort (p = 0.004; Fig 5B). Multiple testing correction using Benjamini-Hochberg methods indicated that the association between L3e and psychomotor speed and dexterity maintained significance, while the relationship between L2a and the executive/working memory composite was not as robust and fell below our p <0.05 cut off for statistical significance.

Because the global deficit score (GDS) is a commonly used tool to assess neurocognitive function, we also examined differences in GDS between the major sub-L haplogroups with at least a 5% present in the cohort; L1b, L1c, L2a, L3b, L3d, L3e and all non-L haplogroups collectively. Results of this analysis showed that there was no difference in GDS between sub-haplogroups (Kruskal-Wallis p-value = 0.572; S1A Fig). When each sub-haplogroup was tested separately as a comparator to the rest of the cohort, there was only a difference in GDS when comparing L2a to all non-L2a individuals (p-value = 0.022), although this association did not remain significant after Benjamini-Hochberg correction (S1B Fig).

### Regression Analysis

To understand and validate the trends in our data, we performed three stepwise linear regressions for each composite score to determine which factors in our cohort best predict neurocognition. In these analyses each composite score was the dependent variable for 3 separate regression models. Independent variables were age, gender, years since therapy started, years since diagnosis, and sub-haplogroup. Sub-haplogroups that represent at least 5% of individuals in the cohort were input as seven separate binary variables L1b, L1c, L2a, L3b, L3d, L3e, and Non-Ls (e.g. 1 meaning the patient is Llb and 0 meaning the patient is not L1b). The best individual predictor for executive/working memory was haplogroup L2a (explaining roughly 5.7% of the variation) and the best overall model also included age and years since diagnosis (explaining roughly 13.1% of the variation) (Table 3). The best individual predictor for declarative memory was age (explaining roughly 3.7% of the variation) and the best overall model also included years since diagnosis (explaining roughly 8.6% of the variation) (Table 3). In contrast, the best individual predictor and overall model for psychomotor speed and dexterity only included haplogroup L3e (explaining roughly 6.1% of the variation) (Table 3). Raw data values from SPSS stepwise linear regression analysis are presented in supplemental information as Table A, B and C in S1 File. These results are consistent with the literature evidence that age and years since diagnosis affecting neurocognitive performance [1, 36] and suggests different sub-haplogroups confer differential sensitivity to motor components of HAND. Additionally, variation in GDS was evaluated using the same step-wise regression parameters and seems to trend similarly to executive/working memory with L2a, age, and years since diagnosis, being the best predictors. These results are presented as suplemental material in S1C Fig.

**Table 3:** SPSS output information from stepwise linear regression.

	Cognitive Components (Dependent Variables)		
	Motor Function	Working Memory	Declarative Memory
**Best individual Predictor**	HapL3e	HapL2a	Age
**Best overall Model**	HapL3e	HapL2a, Age, YrSdiag	Age, YrSdiag
**R Squared (Predictor)**	0.061	0.057	0.037
**R Squared (Model)**	0.061	0.131	0.086
**F value (Predictor)**	9.343	8.843	5.693
**F value (Model)**	9.343	7.334	6.870
**Sig. (Predictor)**	0.003	0.003	0.018
**Sig. (Model)**	0.003	HapL2a: 0.005, Age: 0.001, YrSdiag: 0.030	Age: 0.002, YrSdiag: 0.006
**Beta (Predictor)**	-0.247	0.239	0.193
**Beta (Model)**	-0.247	HapL2a: 0.221, Age: 0.261, YrSdiag: 0.175	Age: 0.253, YrSdiag: -0.229

Each column in Table 3 represents a separate stepwise liner regression, each using a different neuroscore as the dependent variable. R Squared value, F-value, Significance value and Beta values for each regression are listed in the table. SPSS provides results for the best individual predictor as well as the best overall model (a combination of variables that explain the greatest amount of variation in the dependent variable). Independent variables included in each regression include: Age, gender, time since HIV diagnosis, time since CART therapy and sub-haplogroup.

### Further L3e Analysis

**Fig 6:**
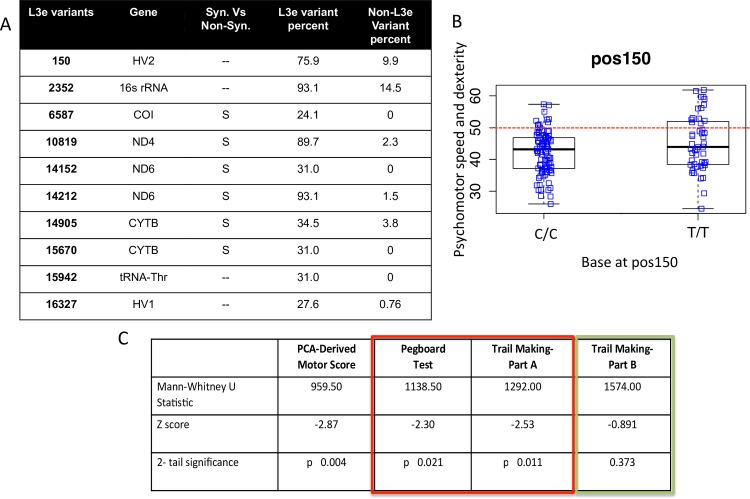
Variants overrepresented in the L3e sub-haplogroup showing association with psychomotor speed and dexterity. A) Fisher’s exact test was used to determine the top 10 variants that were overrepresented in the L3e sub-haplogroup (Fishers exact: p-value < 0.001). Table shows variant, the gene containing the variant, S- synonymous amino acid change vs NS- non-synonymous amino acid change and percent of individuals in L3e or non-L3e who possess each variant. B) Dot plot showing individuals motor score with or without the variant at position 150 (./. reference base in RCRS). Red dotted line indicates mean values of the SENAS control population for the T-scores. C) Individual test scores, which, comprise the PCA derived motor score, were individually evaluated between L3e and non-L3e individuals. Red box indicates simple motor scores, Green box represents complex motor score.

Due to the associations found between individuals of L3e sub-haplogroups and psychomotor speed, we next tested whether there were specific SNPs associated with psychomotor and dexterity performance (Fig 6). In order to determine which variants were overrepresented in L3e individuals we used a Fisher’s exact testing method and filtered for variants that were significantly overrepresented in patients of the L3e haplogroups compared to non-L3e patients. Of the variant positions that are over-represented in the L3e sub-haplogroups of our cohort, positions 150, 10819, 14212 and 14905 were also positively correlated with the psychomotor speed and dexterity composite score (Fig 6A). The 150 variant is particularly interesting due to the fact that it lies in the control region of the mtDNA, which is a region of the mitochondrial genome critical for both transcription and DNA synthesis. Although variants in this region are highly recurrent in multiple haplogroups, they have been shown to correlate with a variety of phenotypes [37] and in our cohort individuals who have the C150T variant have significantly higher motor scores (p = 0.045) (Fig 6B). The other variants over-represented in the L3e subgroups correlated with the psychomotor speed and dexterity composite score were synonymous changes relative to the rCRS mitochondrial reference, presumably having little effect on the physiological function of the protein, although an impacts on mRNA stability or translation efficiency are possible.

### Analysis of Specific Psychomotor Tasks

To better understand the association between the L3e haplogroup and psychomotor speed and dexterity, we analyzed each of the three individual variables that comprised this score and looked for differences between L3e and non-L3e individuals. This found no significant difference in performance on the Trail Making Test- Part B, a test that assesses the capacity to maintain complex mental set (p-value = 0.373; Fig 6C). By contrast, significant differences between L3e and non-L3e were seen in time to completion from both the Grooved Pegboard and Trail Making Test- Part A, tests that assess dexterity and psychomotor speed (p-values = 0,021 and 0,011 respectively; Fig 6C).

### Association of All Variants

Due to the fact that individual variants were associated with differences in neurocognition, we set out to correlate all individual variants with the three neurocognitive-scores. We first determined which positions in our variant table were perfectly correlated with one another, that is to say when there is a variant at one position, there was always a variant at another position. This reduced the number of distinguishable genotypes from 980 to 659. Using logistic regression with the 659 positions and the three neuropsychological composite scores, no significant positions survived Benjimini-Hochberg MTC, although there were several interesting trends (S1 Table). Additionally, heteroplasmy across the mitochondrial DNA was evaluated and the number of heteroplasmic sites per patient were calculated but these values did not seem to correlate with neurocognitive score (Data not shown).

### Analysis of Associations Between Neurocognitive Function and Sub-haplogroups Excluding Non-L Subjects

Racial differences may confound associations between phenotype data and mitochondrial haplogroups. However, the majority of non-L patients in our cohort indicated an African American ancestry, suggesting that their racial backgrounds were similar to the L patients. Nonetheless, we performed an analysis of the data using only patients within the L haplogroup to determine whether the associations identified in the entire cohort would also be detected among subjects with an L haplogroup. This analysis revealed the same associations between L2a and L3e and executive function/working memory and psychomotor speed and dexterity as identified in the larger cohort, although in this case the association between L3e and psychomotor speed and dexterity was not significant following correction for multiple comparisons (S2 Fig, Table A, B and C in S2 File, and S2 Table).

## Discussion

Mitochondrial haplogroups are thought to represent functionally distinct evolutionarily selected populations. Multiple studies have demonstrated a complex interaction between the mitochondrial genome and disease progression, suggesting that mitochondrial genetics can alter disease course [15, 38]. The results of our PCA analysis were consistent with the known evolutionary divergence of the L haplogroups. For example, L0 patient samples, which represent the oldest mitochondrial haplogroup arising ~128.2ka [33, 35], grouped separately in the PCA analysis from the remainder of the patient sample. Similarly, the L1 samples, that represent the next group to diverge in evolutionary terms at ~123.8 ka [35, 39], grouped separately as well. The remainder of the patient samples which included L2, L3, and the non-L patient samples, grouped together in this analysis. The L3 lineage is thought to have arisen in the eastern African mitochondrial gene pool approximately 60–70 ka and subsequently diverged into multiple sub-clades including the N and M lineages, which gave rise to all non-L haplogroups outside of Africa. The L3e lineage arose in central African (Sudan region) 35–60 ka and has further diverged into a number of sub-clades L3e1’2’3’4 which have spread across sub-Saharan Africa and now constitute roughly one third of all L3 lineages [33–35, 39].

Persons infected with HIV-1 often present with motor disabilities including performance on tests that assess discrete fine motor operations [40] and there appears to be involvement of the basal ganglia-thalamo-cortico networks [41]. Valcour et al., assessed motor impairment in HIV-1 infection using the Unified Parkinson’s Disease Rating Scale (UPDRS) and found that HIV-1-infected patients presented with bradykinesia, tremor, and reduced distal muscle agility [42]. These results are consistent with the data reported in the current research in that the relationship between the L3e haplogroups and motor tests was confined to tests that required either fine motor agility (e.g., the Pegboard Test) or motor speed (Trail Making- Part A). The Trail Making Test- Part B differs from our other motor tests in that Trails B provides a measure of the executive ability to maintain a complex mental set. The observation that L3e haplogroups dissociates between simple (Pegboard/ Trails A) versus complex (Trails B) suggests some specificity regarding this haplogroup and simple motor operation. Further research is necessary to explore these relationships.

Within the L3e haplogroups, we identified variants that show promising trends with neurocognition that have also been previously reported in the literature. One such variant is the 150 position, which is of particular interest due to its enrichment in centenarians of European descent [43]. Centenarians live to great age evading the onset of normal age-related pathologies, suggesting the presence of longevity assurance mechanisms in these individuals. The 150 position is a recurrent change, which has arisen in several different haplogroups during the evolution of the mitochondrial genome, suggesting that this site may be unstable. However, cybrid analysis utilizing the C150T variant in either a U or J haplogroup background suggested that the C150T produces lower levels of cellular ROS [44]. Lower ROS production in response to either HIV-1 infection or nucleoside reverse transcriptase inhibitor (NRTI) treatment may have long-term consequences in terms of aging and senescence. In the laboratory we have examined the impact of exposure to NRTIs, which are a component of current cART therapy, in human cells and find a significant increase in both mitochondrial and cellular ROS in cells treated with NRTIs leading to activation of unique mitochondrial stress responses and activation of the senescence program [45]. These results suggest that increased levels of mitochondrial ROS in a chronic setting may activate senescence, which is a component of neurodegenerative disorders such as Alzheimer's disease and other neurocognitive impairments [46].

A limitation of this study is the small size of the sub-cohort, which limited our power to detect associations between haplogroup with neurocognitive function such as working memory. Similarly, the cross-sectional design of the study presents limitations, however future longitudinal assessment of neurocognition in this cohort will allow a more detailed analysis of the influence of mitochondrial genetic variation in regards to HAND progression. Despite the limitations, the data suggests that there is an association between motor function and the presence of sub-haplogroup L3e in the cohort.

A recently completed analysis of haplogroup association with HAND in the CHARTER cohort identified Native American haplogroup B as protective within the admixed Hispanic haplogroups although patients carrying the haplogroups demonstrated lower performance overall [18]. Although the CHARTER study contains a relatively large percent of African American patients, no sub-haplogroup analysis has been carried out specifically examining potential associations with motor function. Future work will reveal if the associations identified in our study can be verified in other cohorts. Further analysis for this study includes analyzing trends associated with the occurrence of insertions or deletions, determining if the degree of heteroplasmy present in these individuals effects neurocognition and analysis of nuclear genes with mitochondrial functions. Together this study will further characterize the role mitochondria may play in the progression of HAND.

## Supporting Information

S1 Fig Global deterioration score data. A) Box whisker plot and Kruskal-Wallis statistics of global deterioration score (GDS on ordinate) between patients of all sub-L haplogroups. B) Box-whisker/dot-plot showing a comparison of differences in GDS in L2a individuals vs. non-L2a individuals. C) Stepwise liner regression results: independent variables included age, gender, sub-haplogroup, years on cART therapy and years since HIV-diagnosis. Dependent variable is global deterioration score.
(TIF)

S2 Fig Neurocognitive PCA excluding non-L patients. A) Rotated component matrix of SENAS neuropsychological test scores used to determine neuropsychological composite scores. Complied in SPSS. Component 1 = declarative memory component 2 = motor and component 3 = working memory. Red boxes show neuroscore groupings for each component determined by the PCA. B) The nine neurocognitive evaluation scores were compiled in SPSS. Total variance explained by each component is contained under the 3 columns listed under Initial Eigenvalues. Components identified as explaining the maximum amount of variance in the data are listed under Extraction Sums of Squared Loadings. Total amount of variance in the model explained by each of these 3 components after varimax rotation is listed under Rotation Sum of Squared Loadings. Total variance explained by the PCA and variance explained by each component of the PCA is outlined in red boxes. C) Histograms showing the three composite scores were normally distributed.
(TIF)

S1 File SPSS tables of linear stepwise regression including all patients. Stepwise linear regressions were performed in SPSS. Independent variables included age, gender, 7 sub-haplogroups that met the 5% threshold cut off (binary variables: L1b, L1c, L2a, L3b, L3d, L3e, non-Ls), years on cART therapy and years since HIV-diagnosis. The three regressions are displayed in separate tables for each dependent variable/neuroscore. **A) Psychomotor speed.** Model summary displaying the best predictor for psychomotor speed as identified by SPSS in terms of R squared value. ANOVA table showing significance for the model. Coefficient table representing Beta values for the model. **B) Executive/working memory.** Model summary displaying the best predictors for working memory as identified by SPSS in terms of R squared value. ANOVA table showing significance for each model. Coefficient table representing Beta values for each model. **C) Declarative memory.** Model summary displaying the best predictors for declarative memory as identified by SPSS in terms of R squared value. ANOVA table showing significance for each model. Coefficient table representing Beta values for each model.
(ZIP)

S2 File Linear regression analysis excluding non-L patients. Independent variables included Age, Gender, 6 sub-haplogroups that met the 5% threshold cut off (binary variables: L1b, L1c, L2a, L3b, L3d and L3e), years on cART therapy and years since HIV-diagnosis. Three regressions displayed in separate tables for each dependent variable/neuroscore **A) Psychomotor speed.** Model summary displaying the best predictor for psychomotor speed as identified by SPSS in terms of R squared value. ANOVA table showing significance for the model. Coefficient table representing Beta values for the model. **B) Executive/working memory.** Model summary displaying the best predictors for working memory as identified by SPSS in terms of R squared value. ANOVA table showing significance for each model. Coefficient table representing Beta values for each model. **C) Declarative memory.** Model summary displaying the best predictors for declarative memory as identified by SPSS in terms of R squared value. ANOVA table showing significance for each model. Coefficient table representing Beta values for each model.
(ZIP)

S1 Table Individual variants associated with neuroscore. All variants in the cohort were analyzed using logistic regression to determine association with the three-neurocognitive composite scores. Table contains the nucleotide positions that associated with differences in each Neurocognitive Composite Score. (raw p < 0.05, not multiple testing corrected).
(TIF)

S2 Table Sub-haplogroup associations with neurocognitive performance when segregated from the cohort as a whole excluding non-L patients. Specific sub-haplogroups are listed with a breakdown including number of patients of each sub-haplogroup and the p-values for all Mann-Whitney U segregation comparisons for the three composite neuroscores derived from the PCA analysis as well as p-values for comparisons with GDS. Total number of patients listed is less than total number of patients in Table 1 due to missing neurocognitive values. Sub-haplogroups, which contain fewer than 5% of the total cohort, were excluded from the analysis.
(TIF)

S3 Table List of haplogroup information for all patients. Patient ID (Patient unique identifier), Haplofind (full haplogroup assignment from Haplofind website), MajorHap (Major Haplogroup i.e. first 2 characters of the full haplogroup assignment), MajorHap Class (Categorical groupings of Major Haplogroups for statistical analysis), Subhap (Sub Haplogroup i.e. first 3 characters of the full haplogroup assignment, Subhap Class (Categorical groupings of Major Haplogroups for statistical analysis).
(ZIP)

S4 Table SRA BioSample Information. Table containing accession numbers for the submission of two fastq read files for each patient (paired end reads) submitted to NCBI Sequence read archive BioProject number PRJNA321053 SRA accession: SRP074574.
(ZIP)
