# Supplementary figures and images for: Mitochondrial Haplogroup Influences Motor Function in Long-Term HIV-1-Infected Individuals
Source: PLoS One. 2016 Oct 6;11(10):e0163772. doi: 10.1371/journal.pone.0163772 (PMC5053473; doi:10.1371/journal.pone.0163772)

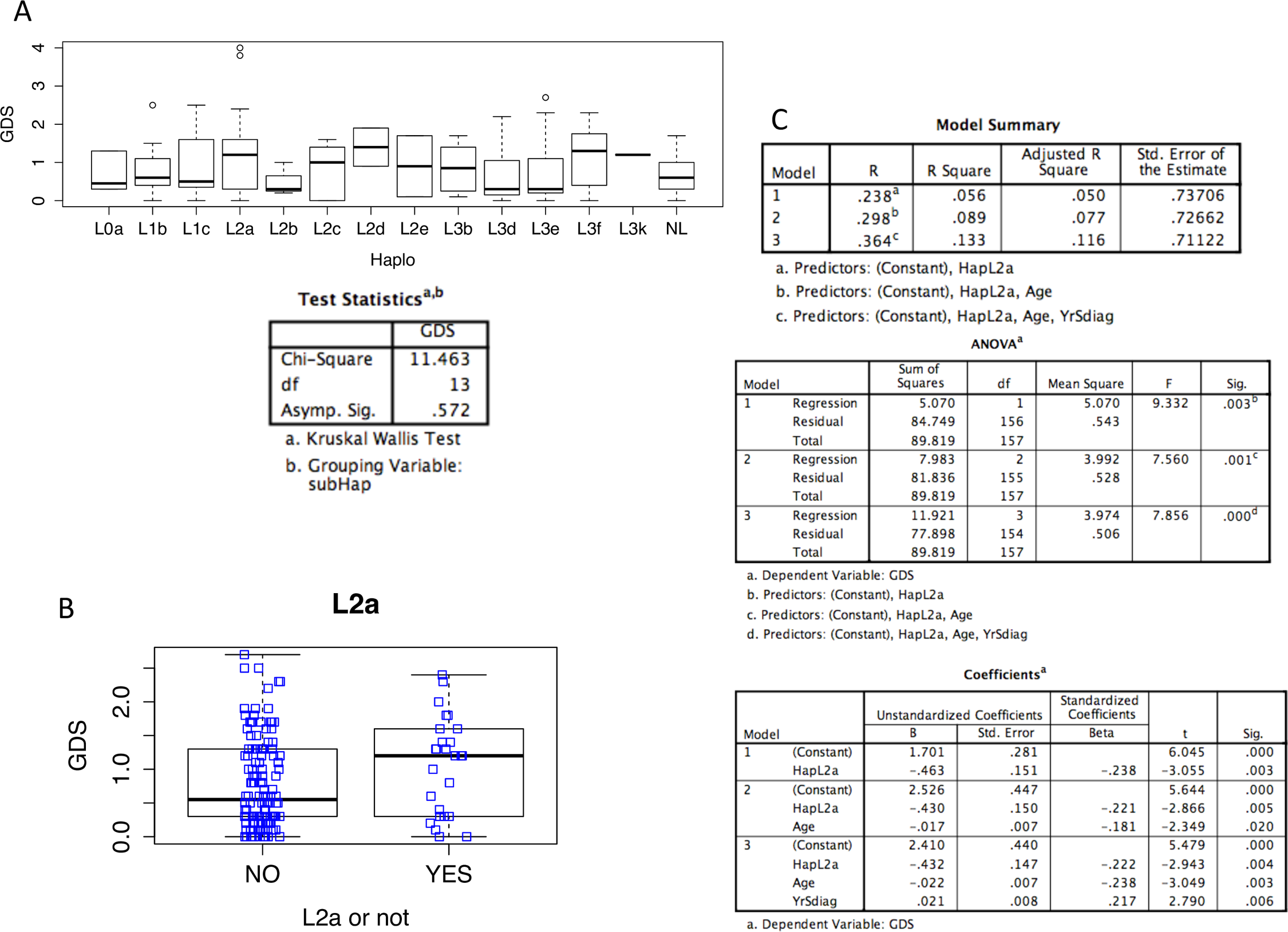

Supplement: S1 Fig — A) Box whisker plot and Kruskal-Wallis statistics of global deterioration score (GDS on ordinate) between patients of all sub-L haplogroups. B) Box-whisker/dot-plot showing a comparison of differences in GDS in L2a individuals vs. non-L2a individuals. C) Stepwise liner regression results: independent variables included age, gender, sub-haplogroup, years on cART therapy and years since HIV-diagnosis. Dependent variable is global deterioration score. (TIF) [file pone.0163772.s001.tif]

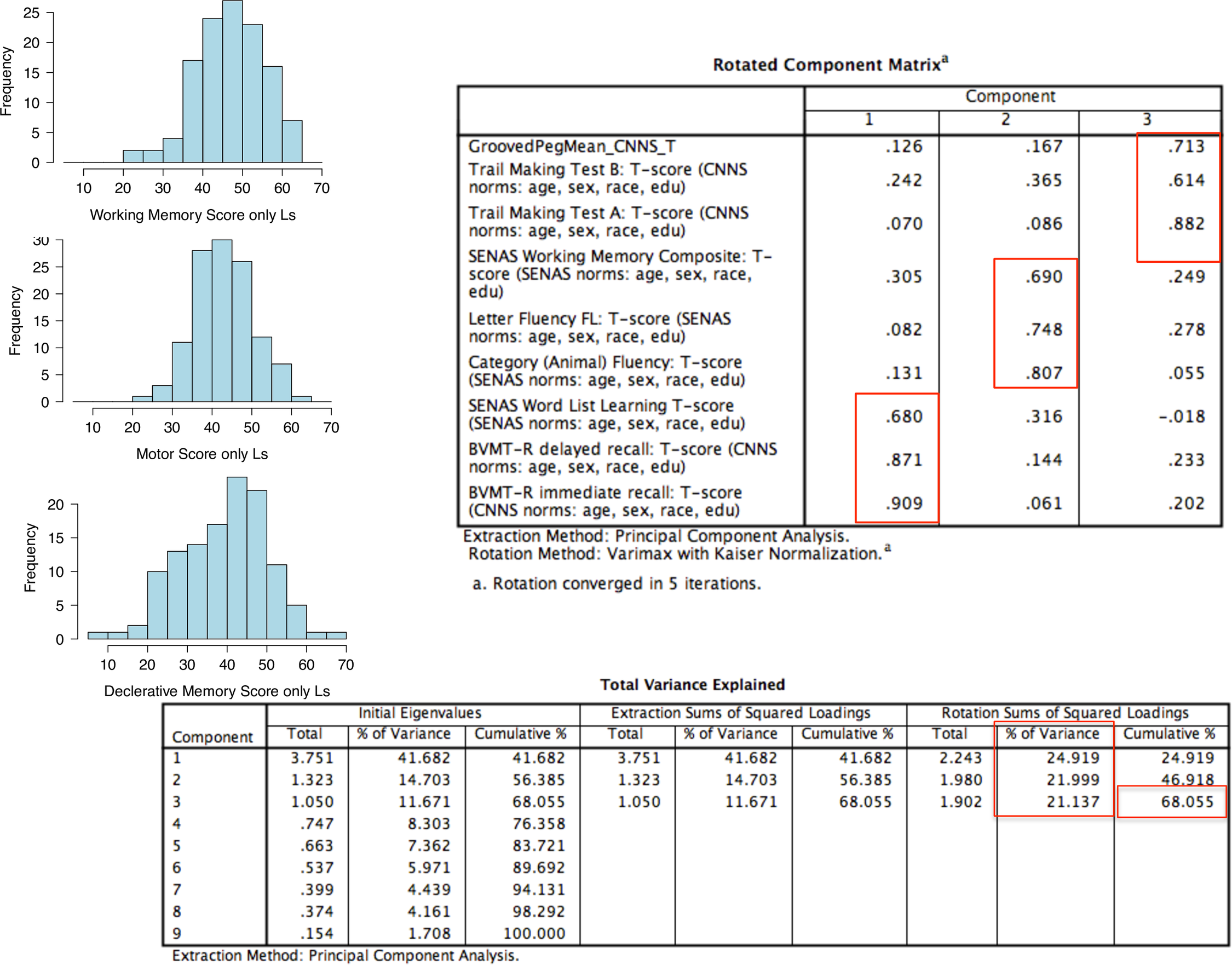

Supplement: S2 Fig — A) Rotated component matrix of SENAS neuropsychological test scores used to determine neuropsychological composite scores. Complied in SPSS. Component 1 = declarative memory component 2 = motor and component 3 = working memory. Red boxes show neuroscore groupings for each component determined by the PCA. B) The nine neurocognitive evaluation scores were compiled in SPSS. Total variance explained by each component is contained under the 3 columns listed under Initial Eigenvalues. Components identified as explaining the maximum amount of variance in the data are listed under Extraction Sums of Squared Loadings. Total amount of variance in the model explained by each of these 3 components after varimax rotation is listed under Rotation Sum of Squared Loadings. Total variance explained by the PCA and variance explained by each component of the PCA is outlined in red boxes. C) Histograms showing the three composite scores were normally distributed. (TIF) [file pone.0163772.s002.tif]

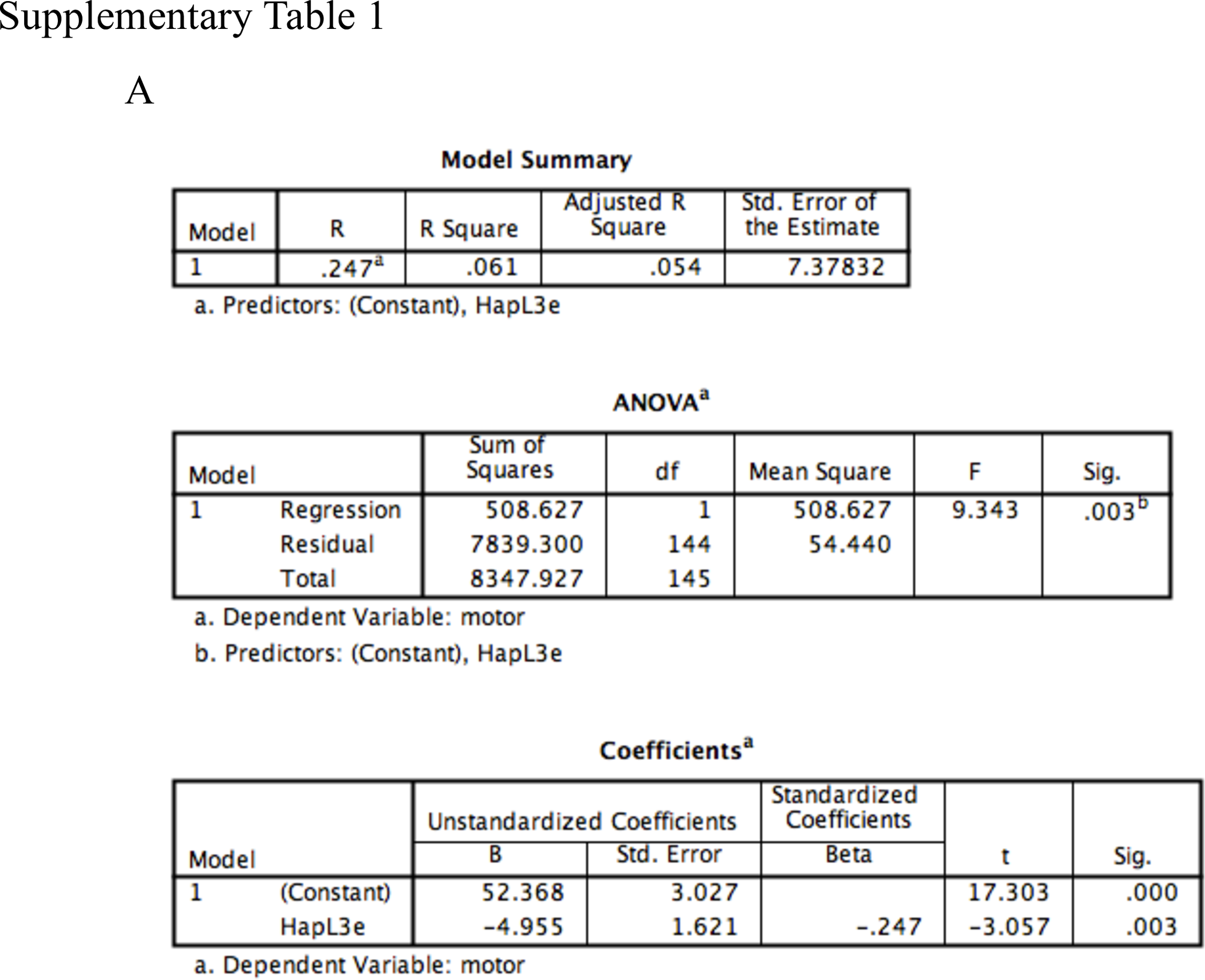

Supplement: S1 File — Stepwise linear regressions were performed in SPSS. Independent variables included age, gender, 7 sub-haplogroups that met the 5% threshold cut off (binary variables: L1b, L1c, L2a, L3b, L3d, L3e, non-Ls), years on cART therapy and years since HIV-diagnosis. The three regressions are displayed in separate tables for each dependent variable/neuroscore. A) Psychomotor speed. Model summary displaying the best predictor for psychomotor speed as identified by SPSS in terms of R squared value. ANOVA table showing significance for the model. Coefficient table representing Beta values for the model. B) Executive/working memory. Model summary displaying the best predictors for working memory as identified by SPSS in terms of R squared value. ANOVA table showing significance for each model. Coefficient table representing Beta values for each model. C) Declarative memory. Model summary displaying the best predictors for declarative memory as identified by SPSS in terms of R squared value. ANOVA table showing significance for each model. Coefficient table representing Beta values for each model. (ZIP) [file pone.0163772.s003.zip › supptable1A.tif]

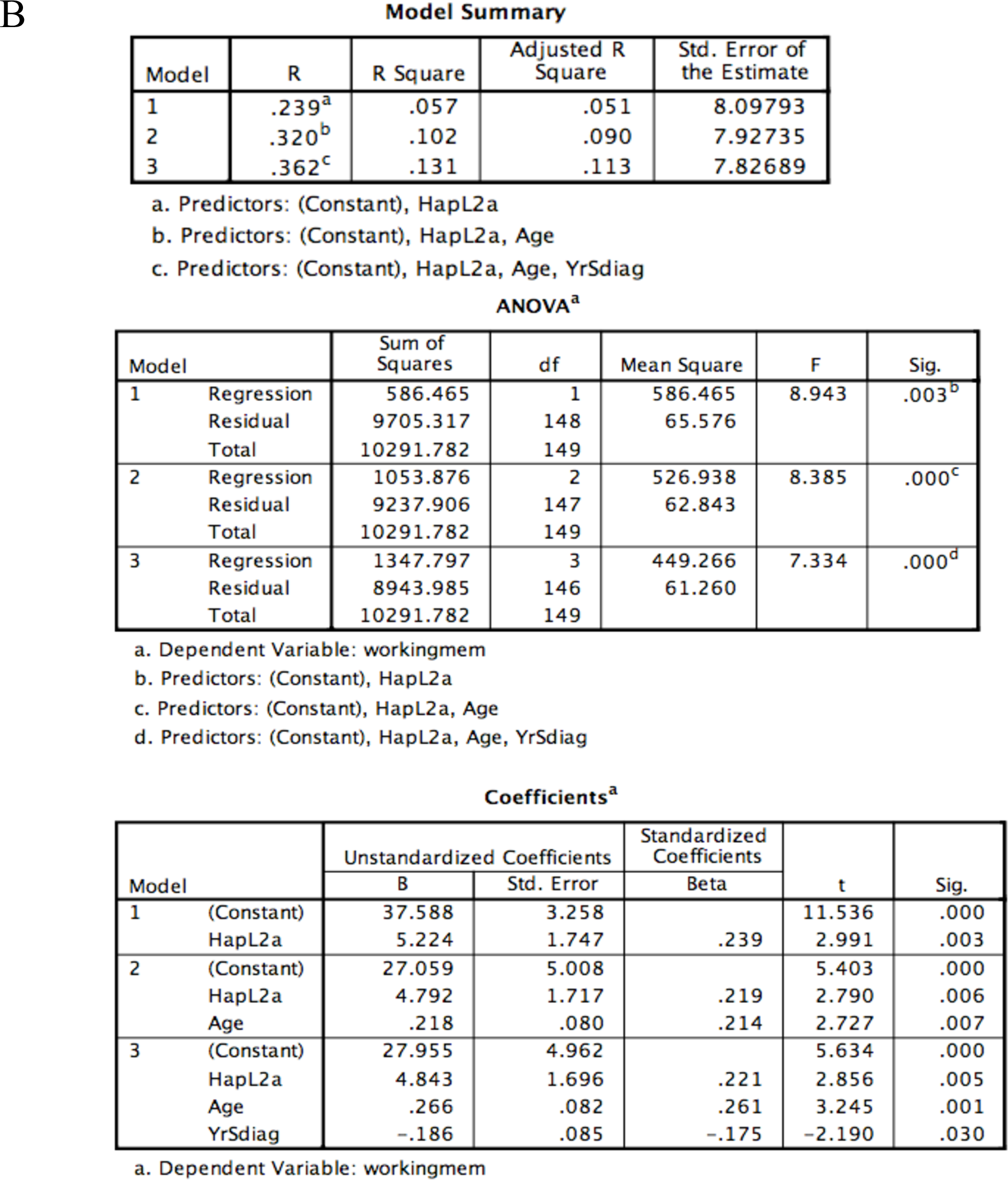

Supplement: S1 File — Stepwise linear regressions were performed in SPSS. Independent variables included age, gender, 7 sub-haplogroups that met the 5% threshold cut off (binary variables: L1b, L1c, L2a, L3b, L3d, L3e, non-Ls), years on cART therapy and years since HIV-diagnosis. The three regressions are displayed in separate tables for each dependent variable/neuroscore. A) Psychomotor speed. Model summary displaying the best predictor for psychomotor speed as identified by SPSS in terms of R squared value. ANOVA table showing significance for the model. Coefficient table representing Beta values for the model. B) Executive/working memory. Model summary displaying the best predictors for working memory as identified by SPSS in terms of R squared value. ANOVA table showing significance for each model. Coefficient table representing Beta values for each model. C) Declarative memory. Model summary displaying the best predictors for declarative memory as identified by SPSS in terms of R squared value. ANOVA table showing significance for each model. Coefficient table representing Beta values for each model. (ZIP) [file pone.0163772.s003.zip › supptable1B.tif]

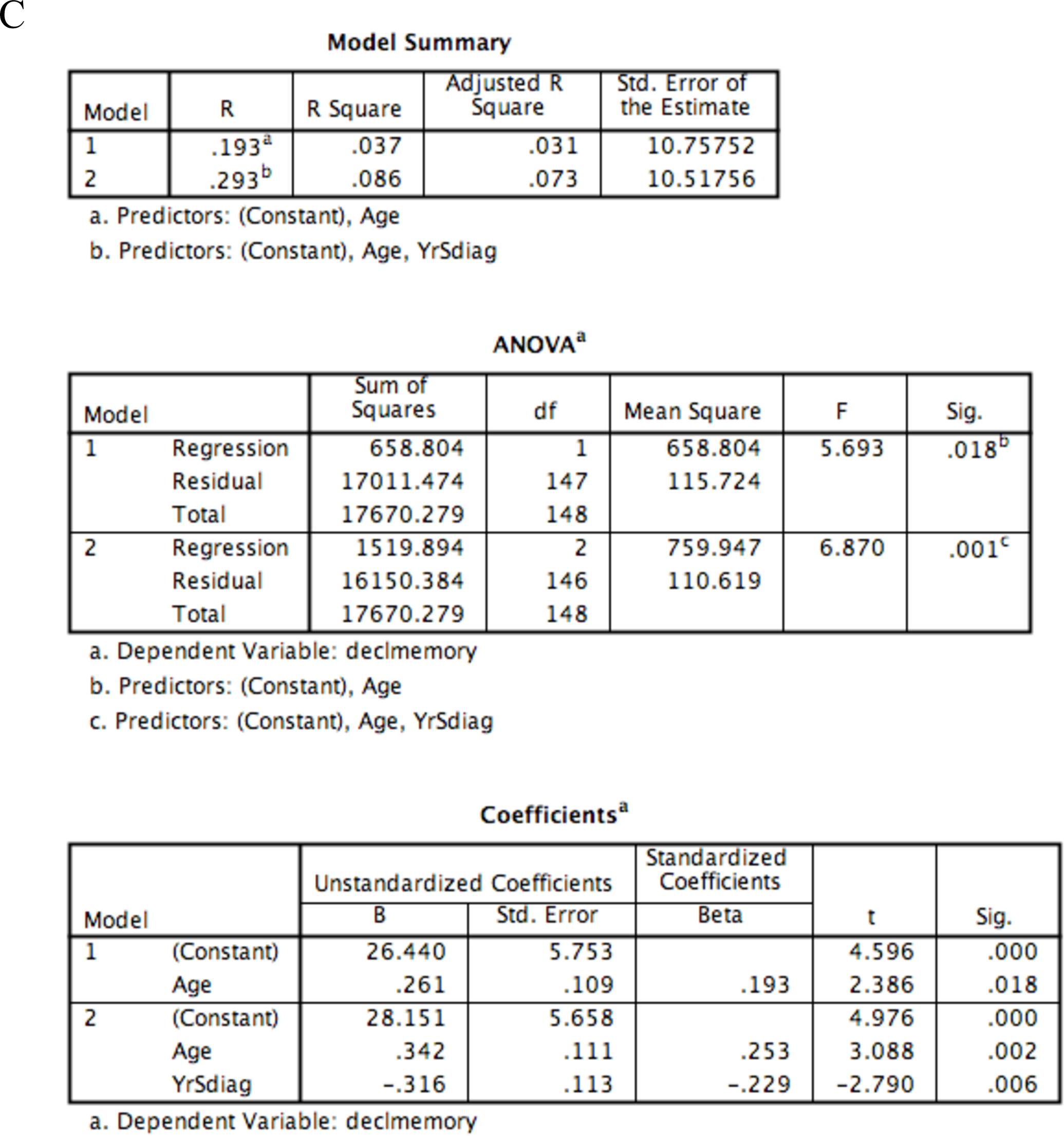

Supplement: S1 File — Stepwise linear regressions were performed in SPSS. Independent variables included age, gender, 7 sub-haplogroups that met the 5% threshold cut off (binary variables: L1b, L1c, L2a, L3b, L3d, L3e, non-Ls), years on cART therapy and years since HIV-diagnosis. The three regressions are displayed in separate tables for each dependent variable/neuroscore. A) Psychomotor speed. Model summary displaying the best predictor for psychomotor speed as identified by SPSS in terms of R squared value. ANOVA table showing significance for the model. Coefficient table representing Beta values for the model. B) Executive/working memory. Model summary displaying the best predictors for working memory as identified by SPSS in terms of R squared value. ANOVA table showing significance for each model. Coefficient table representing Beta values for each model. C) Declarative memory. Model summary displaying the best predictors for declarative memory as identified by SPSS in terms of R squared value. ANOVA table showing significance for each model. Coefficient table representing Beta values for each model. (ZIP) [file pone.0163772.s003.zip › supptable1C.tif]

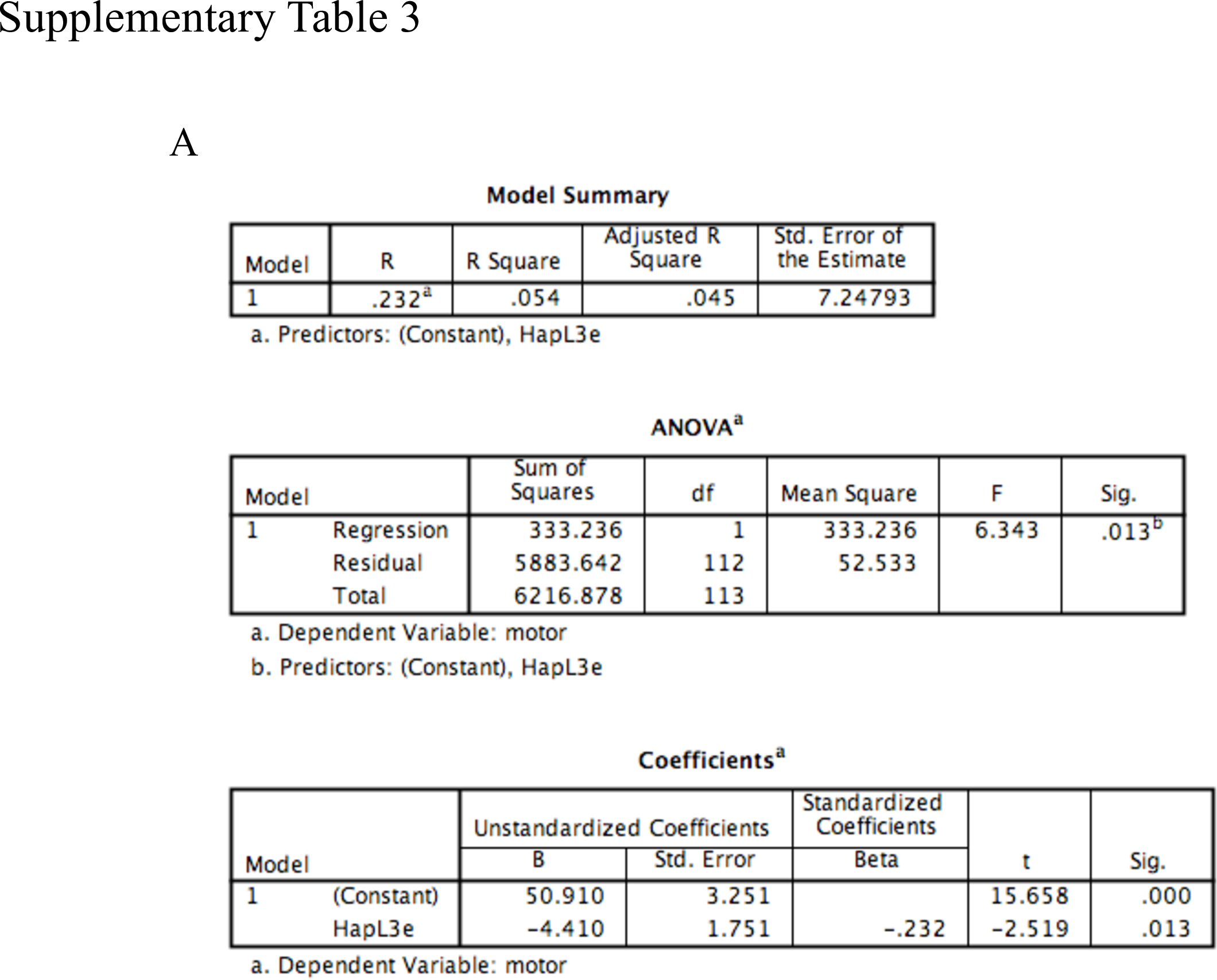

Supplement: S2 File — Independent variables included Age, Gender, 6 sub-haplogroups that met the 5% threshold cut off (binary variables: L1b, L1c, L2a, L3b, L3d and L3e), years on cART therapy and years since HIV-diagnosis. Three regressions displayed in separate tables for each dependent variable/neuroscore A) Psychomotor speed. Model summary displaying the best predictor for psychomotor speed as identified by SPSS in terms of R squared value. ANOVA table showing significance for the model. Coefficient table representing Beta values for the model. B) Executive/working memory. Model summary displaying the best predictors for working memory as identified by SPSS in terms of R squared value. ANOVA table showing significance for each model. Coefficient table representing Beta values for each model. C) Declarative memory. Model summary displaying the best predictors for declarative memory as identified by SPSS in terms of R squared value. ANOVA table showing significance for each model. Coefficient table representing Beta values for each model. (ZIP) [file pone.0163772.s004.zip › supptable3A.tif]

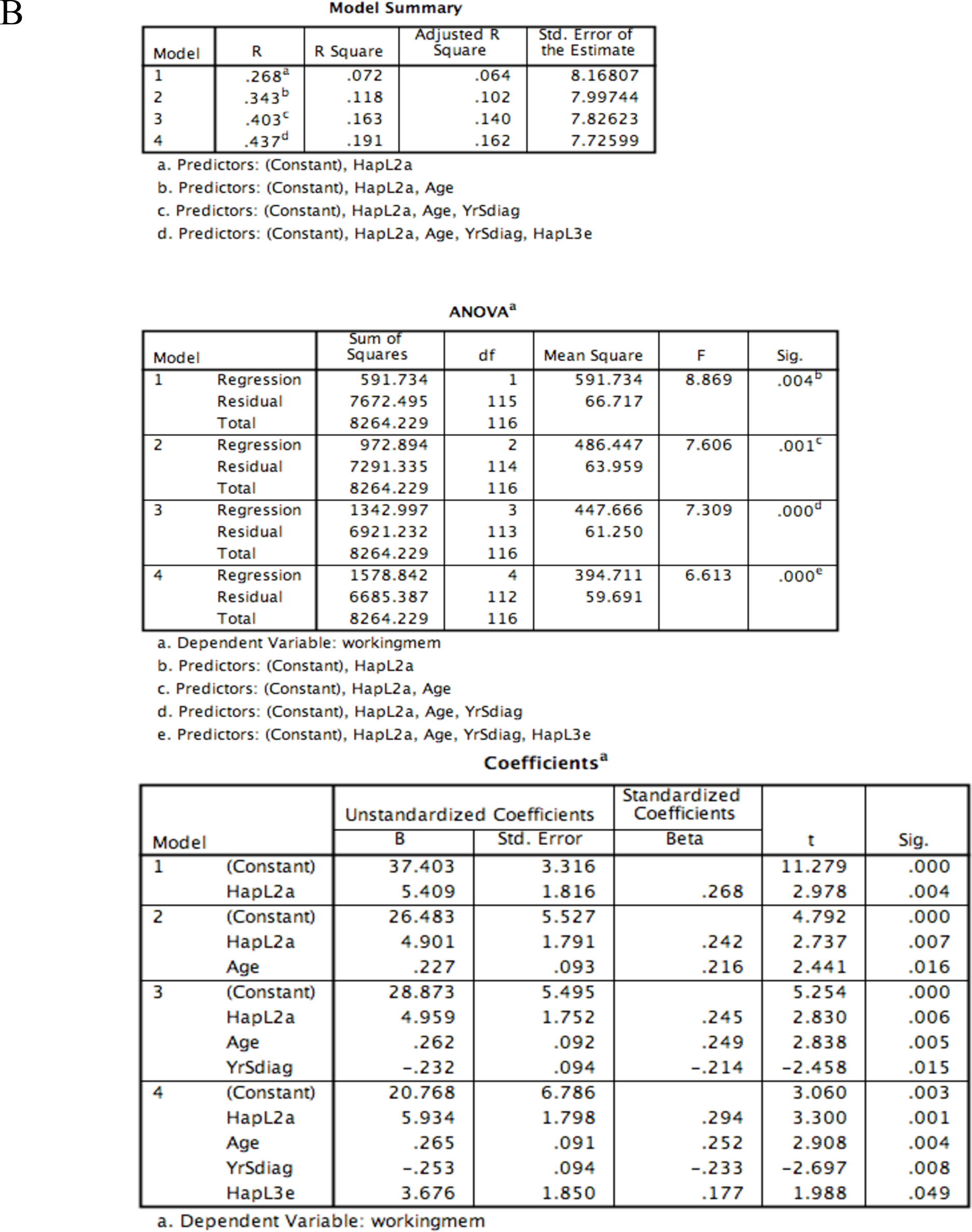

Supplement: S2 File — Independent variables included Age, Gender, 6 sub-haplogroups that met the 5% threshold cut off (binary variables: L1b, L1c, L2a, L3b, L3d and L3e), years on cART therapy and years since HIV-diagnosis. Three regressions displayed in separate tables for each dependent variable/neuroscore A) Psychomotor speed. Model summary displaying the best predictor for psychomotor speed as identified by SPSS in terms of R squared value. ANOVA table showing significance for the model. Coefficient table representing Beta values for the model. B) Executive/working memory. Model summary displaying the best predictors for working memory as identified by SPSS in terms of R squared value. ANOVA table showing significance for each model. Coefficient table representing Beta values for each model. C) Declarative memory. Model summary displaying the best predictors for declarative memory as identified by SPSS in terms of R squared value. ANOVA table showing significance for each model. Coefficient table representing Beta values for each model. (ZIP) [file pone.0163772.s004.zip › supptable3B.tif]

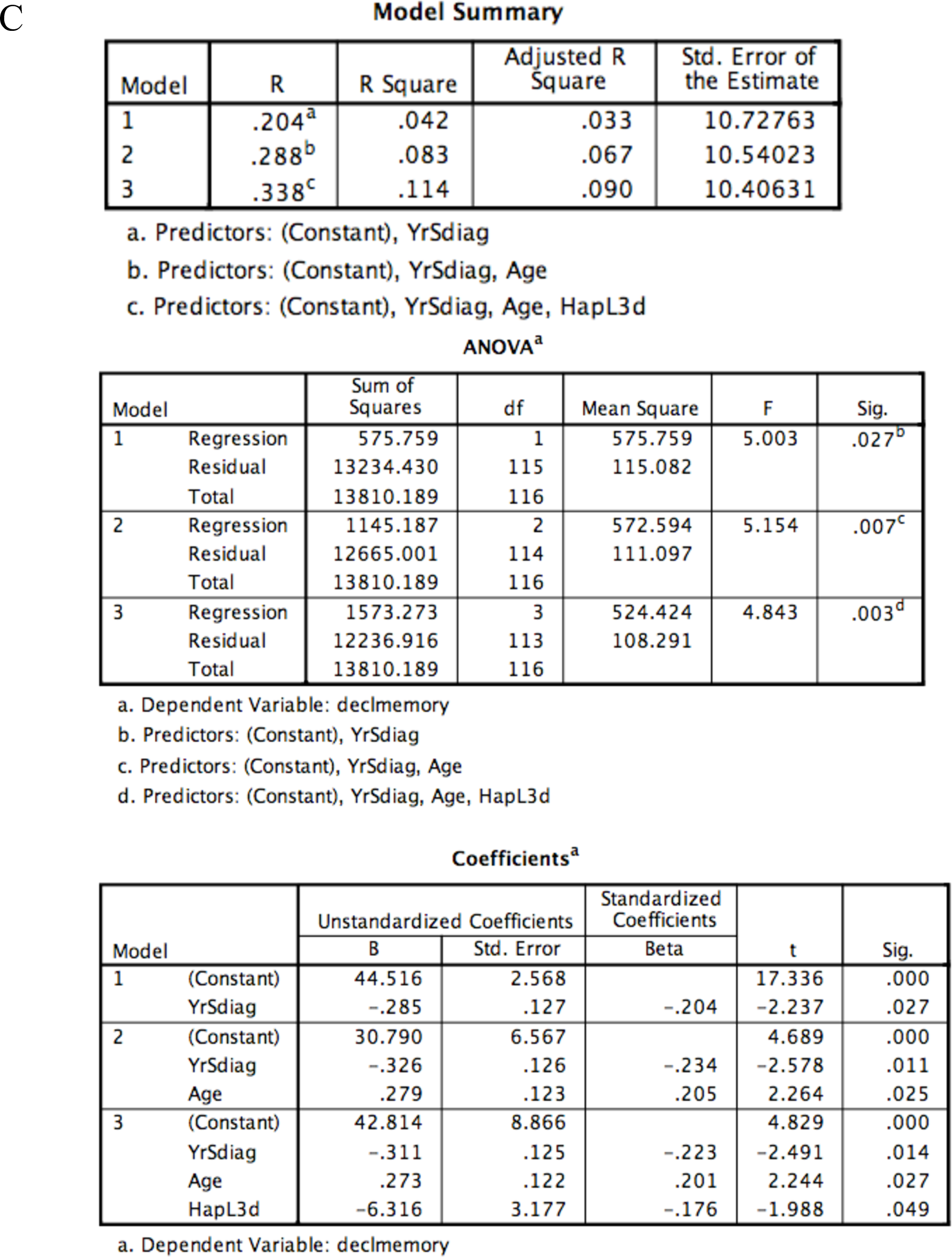

Supplement: S2 File — Independent variables included Age, Gender, 6 sub-haplogroups that met the 5% threshold cut off (binary variables: L1b, L1c, L2a, L3b, L3d and L3e), years on cART therapy and years since HIV-diagnosis. Three regressions displayed in separate tables for each dependent variable/neuroscore A) Psychomotor speed. Model summary displaying the best predictor for psychomotor speed as identified by SPSS in terms of R squared value. ANOVA table showing significance for the model. Coefficient table representing Beta values for the model. B) Executive/working memory. Model summary displaying the best predictors for working memory as identified by SPSS in terms of R squared value. ANOVA table showing significance for each model. Coefficient table representing Beta values for each model. C) Declarative memory. Model summary displaying the best predictors for declarative memory as identified by SPSS in terms of R squared value. ANOVA table showing significance for each model. Coefficient table representing Beta values for each model. (ZIP) [file pone.0163772.s004.zip › supptable3C.tif]

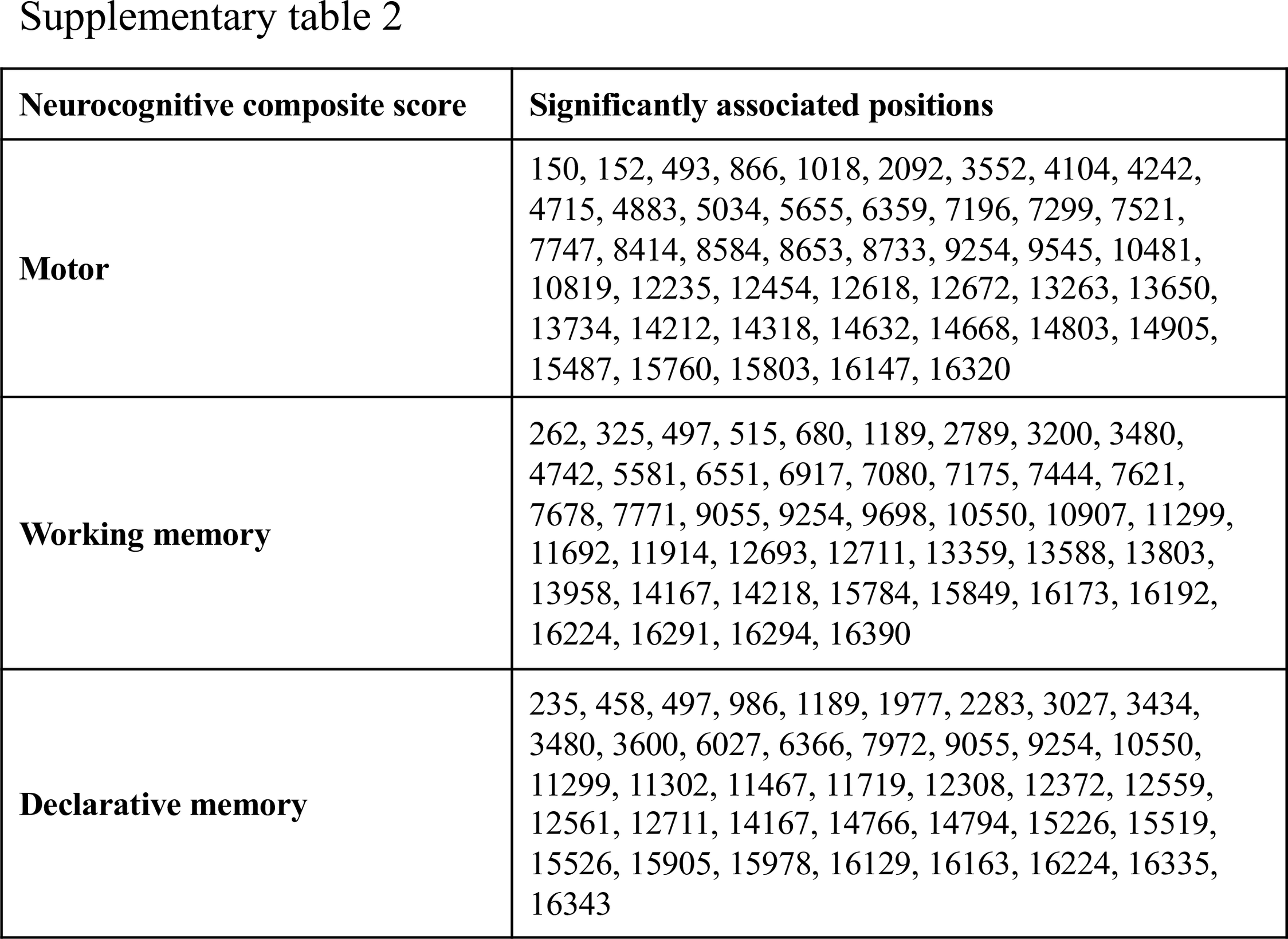

Supplement: S1 Table — All variants in the cohort were analyzed using logistic regression to determine association with the three-neurocognitive composite scores. Table contains the nucleotide positions that associated with differences in each Neurocognitive Composite Score. (raw p < 0.05, not multiple testing corrected). (TIF) [file pone.0163772.s005.tif]

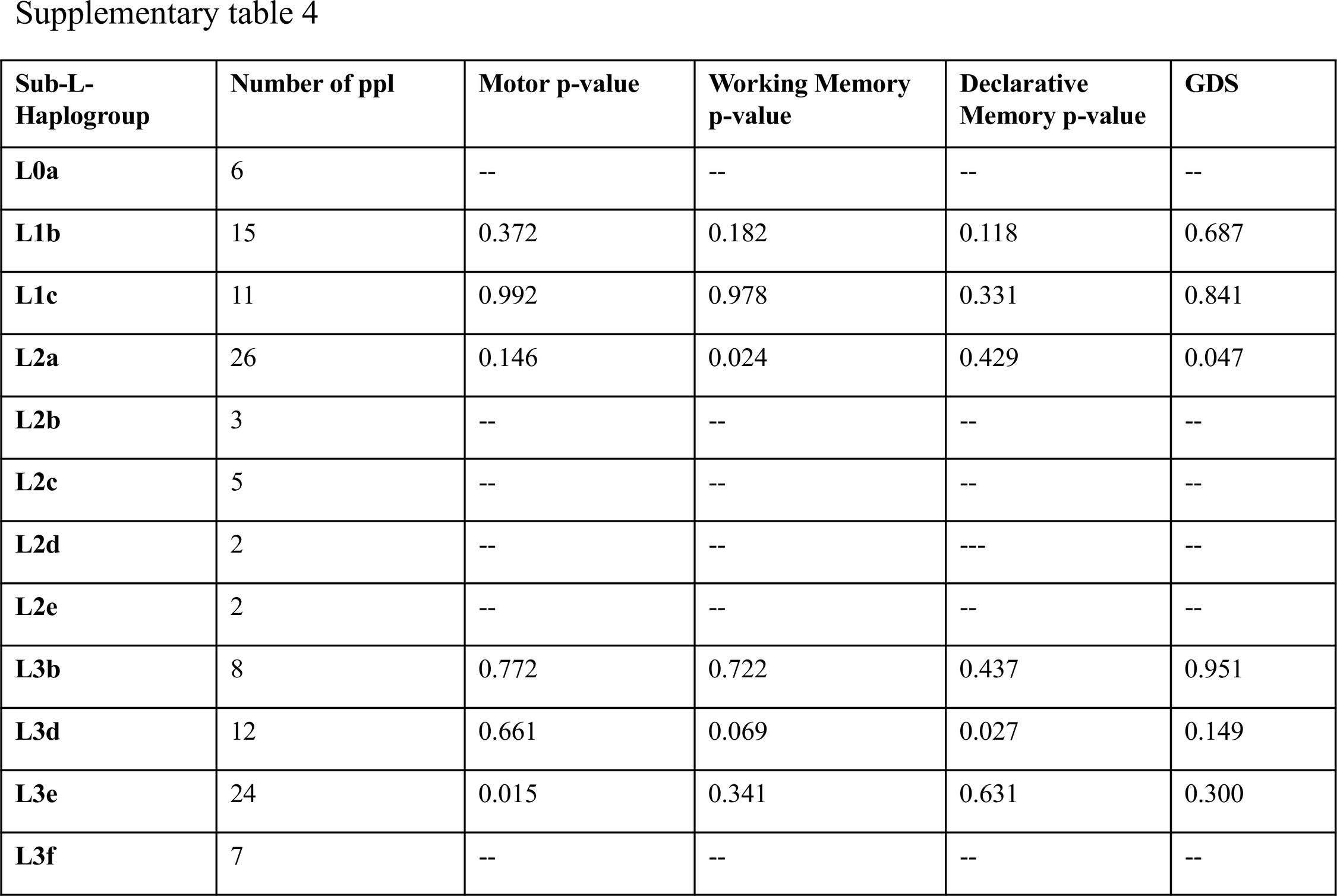

Supplement: S2 Table — Specific sub-haplogroups are listed with a breakdown including number of patients of each sub-haplogroup and the p-values for all Mann-Whitney U segregation comparisons for the three composite neuroscores derived from the PCA analysis as well as p-values for comparisons with GDS. Total number of patients listed is less than total number of patients in Table 1 due to missing neurocognitive values. Sub-haplogroups, which contain fewer than 5% of the total cohort, were excluded from the analysis. (TIF) [file pone.0163772.s006.tif]

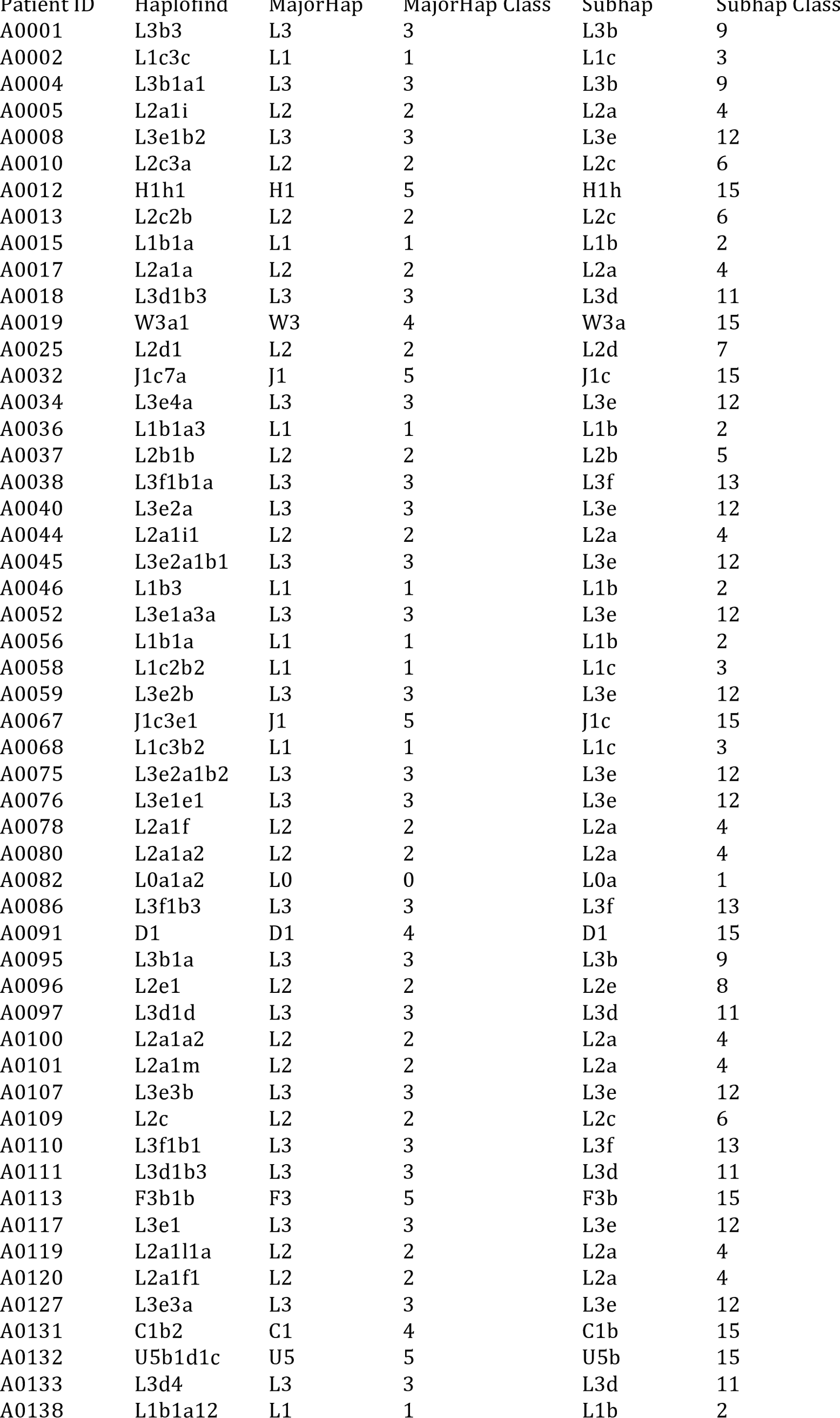

Supplement: S3 Table — Patient ID (Patient unique identifier), Haplofind (full haplogroup assignment from Haplofind website), MajorHap (Major Haplogroup i.e. first 2 characters of the full haplogroup assignment), MajorHap Class (Categorical groupings of Major Haplogroups for statistical analysis), Subhap (Sub Haplogroup i.e. first 3 characters of the full haplogroup assignment, Subhap Class (Categorical groupings of Major Haplogroups for statistical analysis). (ZIP) [file pone.0163772.s007.zip › Supptable5-1.tif]

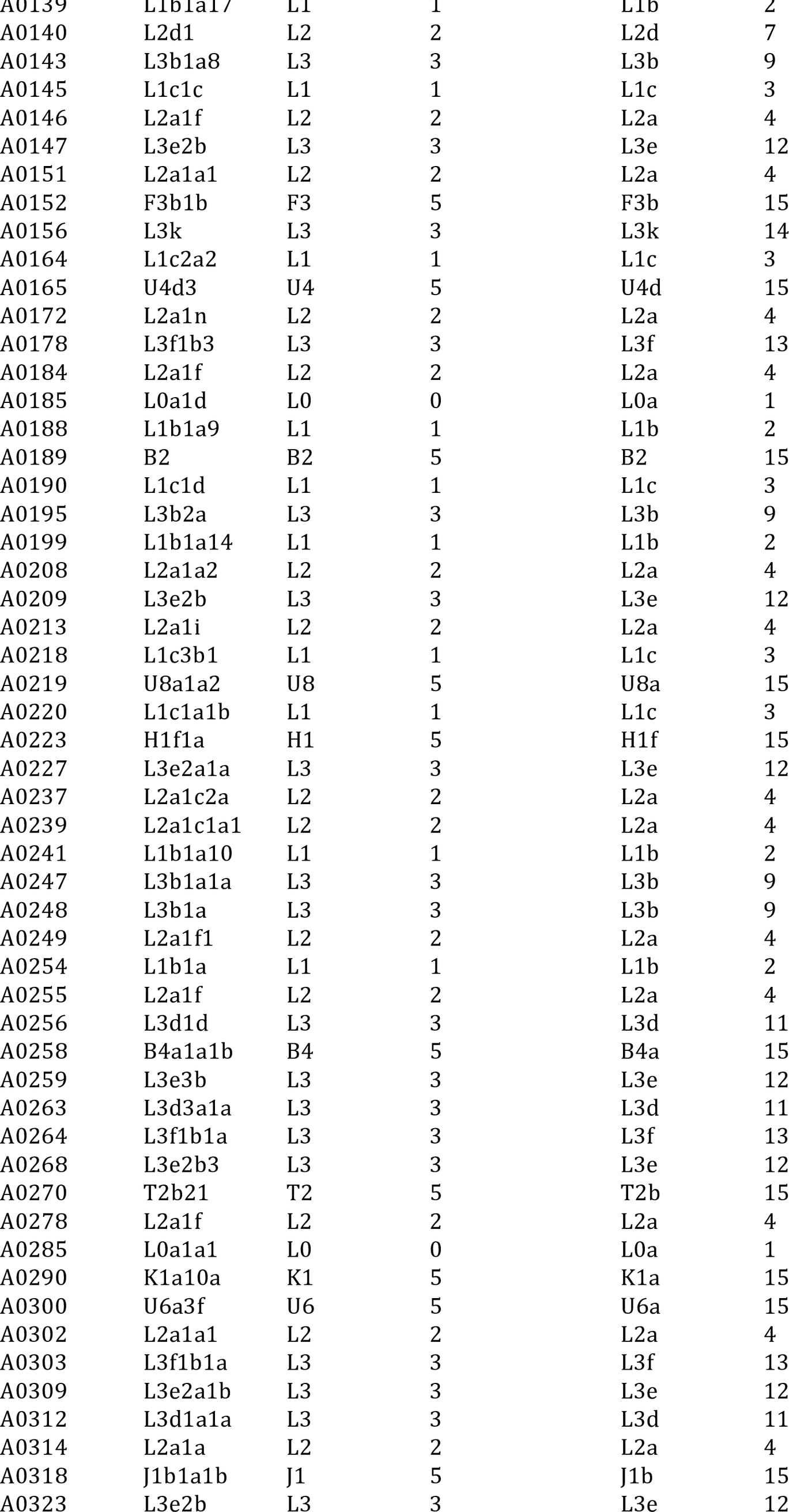

Supplement: S3 Table — Patient ID (Patient unique identifier), Haplofind (full haplogroup assignment from Haplofind website), MajorHap (Major Haplogroup i.e. first 2 characters of the full haplogroup assignment), MajorHap Class (Categorical groupings of Major Haplogroups for statistical analysis), Subhap (Sub Haplogroup i.e. first 3 characters of the full haplogroup assignment, Subhap Class (Categorical groupings of Major Haplogroups for statistical analysis). (ZIP) [file pone.0163772.s007.zip › Supptable5-2.tif]

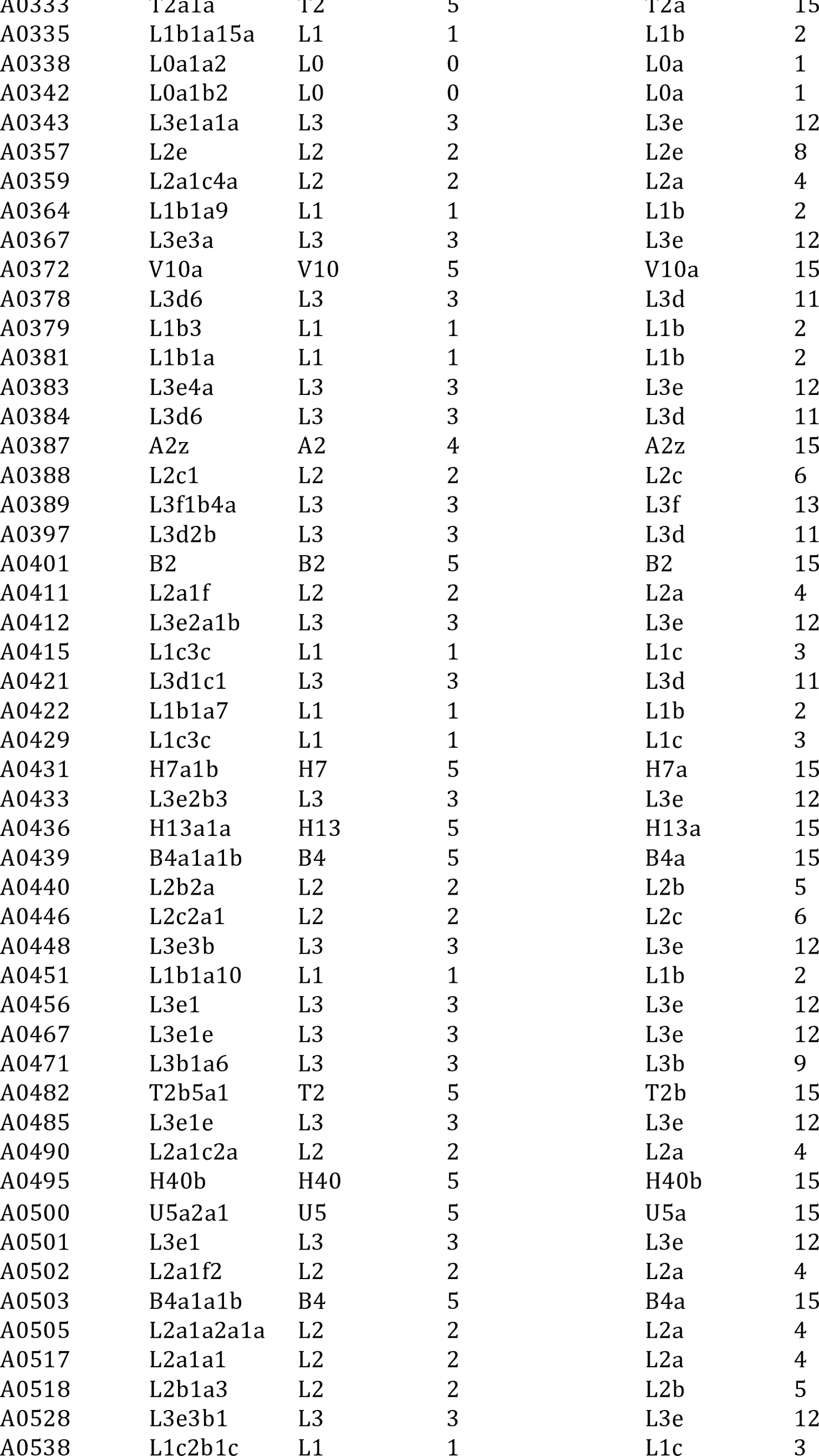

Supplement: S3 Table — Patient ID (Patient unique identifier), Haplofind (full haplogroup assignment from Haplofind website), MajorHap (Major Haplogroup i.e. first 2 characters of the full haplogroup assignment), MajorHap Class (Categorical groupings of Major Haplogroups for statistical analysis), Subhap (Sub Haplogroup i.e. first 3 characters of the full haplogroup assignment, Subhap Class (Categorical groupings of Major Haplogroups for statistical analysis). (ZIP) [file pone.0163772.s007.zip › Supptable5-3.tif]

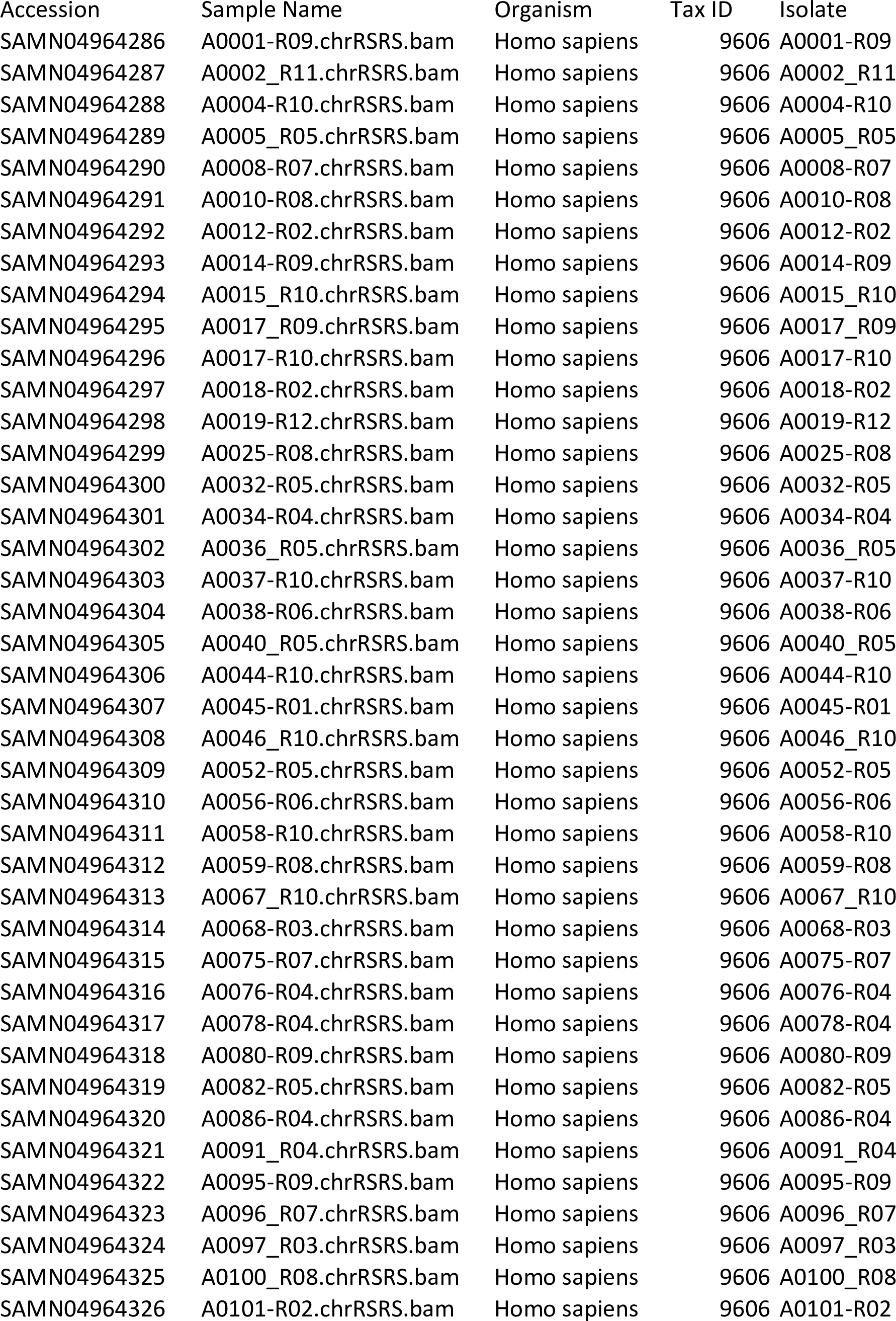

Supplement: S4 Table — Table containing accession numbers for the submission of two fastq read files for each patient (paired end reads) submitted to NCBI Sequence read archive BioProject number PRJNA321053 SRA accession: SRP074574. (ZIP) [file pone.0163772.s008.zip › Supptable6-1.tif]

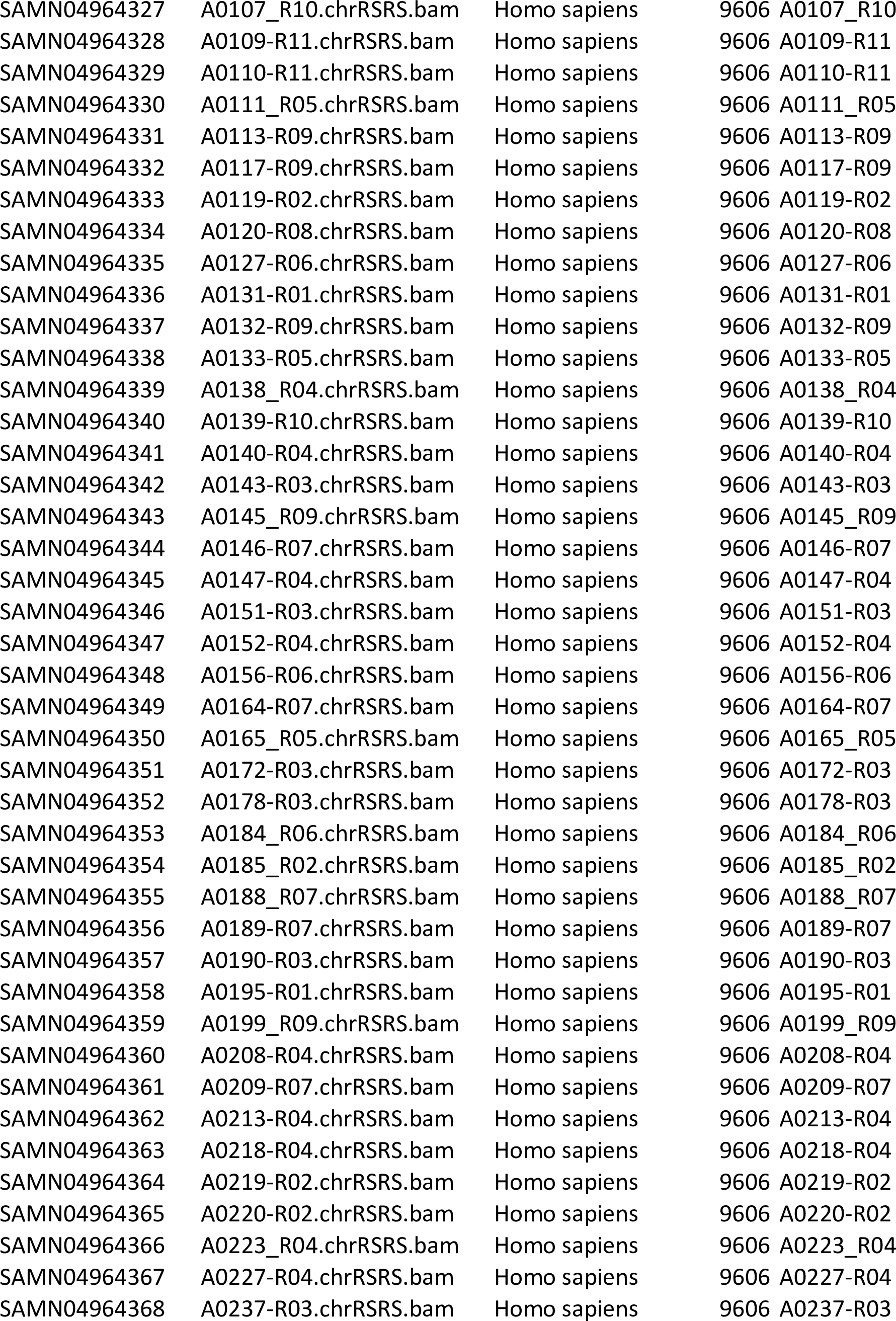

Supplement: S4 Table — Table containing accession numbers for the submission of two fastq read files for each patient (paired end reads) submitted to NCBI Sequence read archive BioProject number PRJNA321053 SRA accession: SRP074574. (ZIP) [file pone.0163772.s008.zip › Supptable6-2.tif]

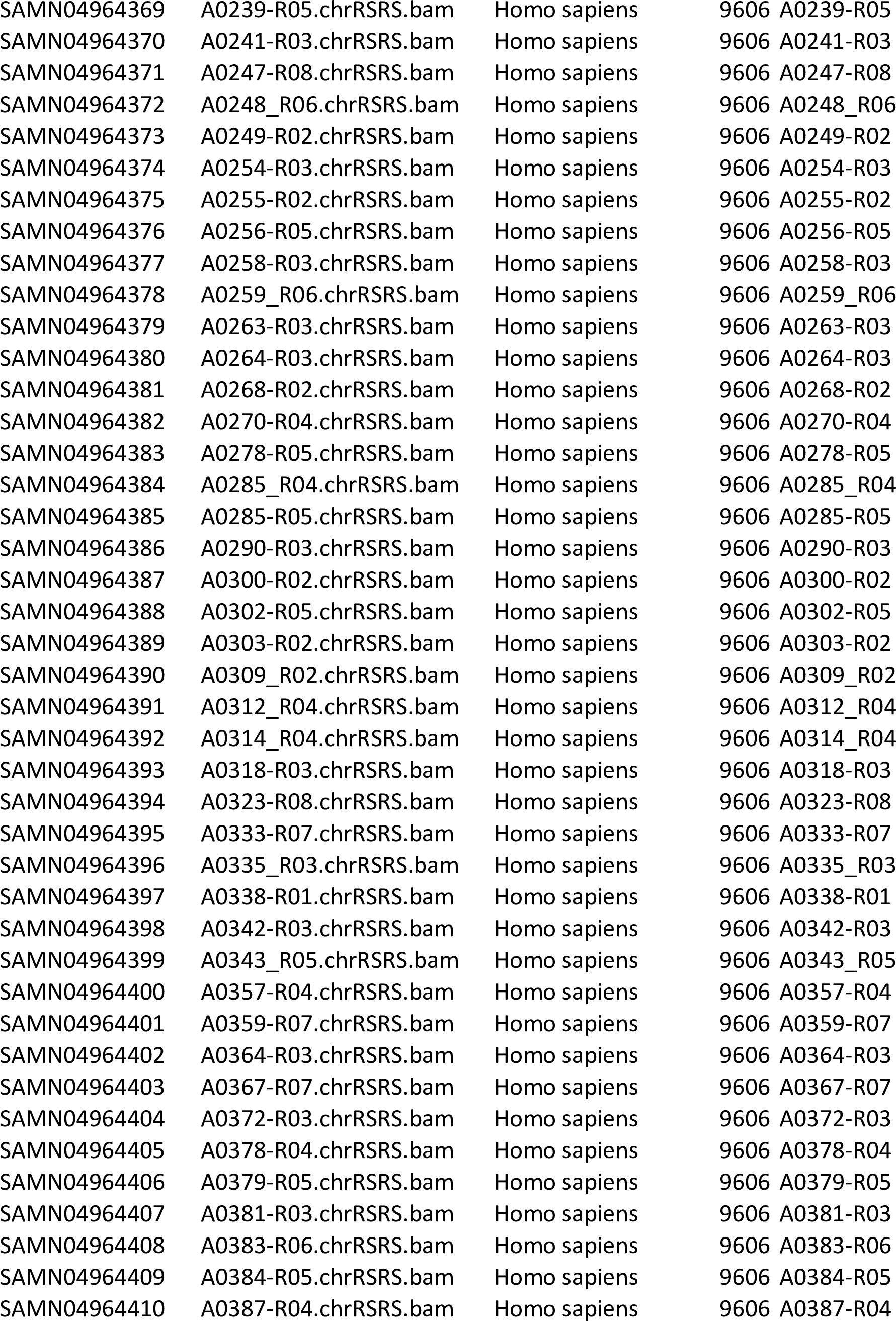

Supplement: S4 Table — Table containing accession numbers for the submission of two fastq read files for each patient (paired end reads) submitted to NCBI Sequence read archive BioProject number PRJNA321053 SRA accession: SRP074574. (ZIP) [file pone.0163772.s008.zip › Supptable6-3.tif]

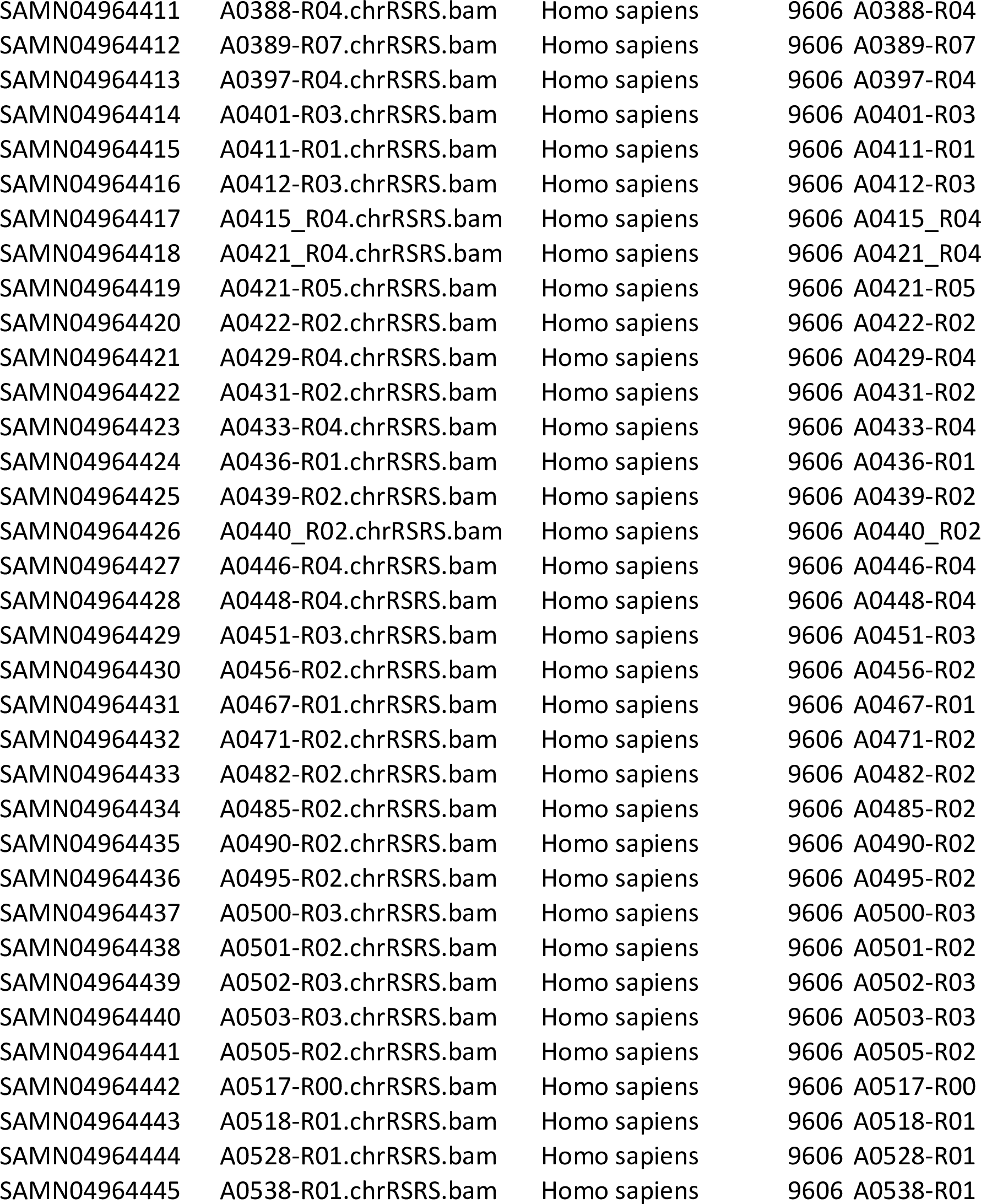

Supplement: S4 Table — Table containing accession numbers for the submission of two fastq read files for each patient (paired end reads) submitted to NCBI Sequence read archive BioProject number PRJNA321053 SRA accession: SRP074574. (ZIP) [file pone.0163772.s008.zip › Supptable6-4.tif]
